# Oxidation of Disulfides to Thiolsulfinates with Hydrogen Peroxide and a Cyclic Seleninate Ester Catalyst

**DOI:** 10.3390/molecules200610748

**Published:** 2015-06-11

**Authors:** Nicole M. R. McNeil, Ciara McDonnell, Miranda Hambrook, Thomas G. Back

**Affiliations:** Department of Chemistry, University of Calgary, Calgary, AB T2N 1N4, Canada; E-Mails: nmrmcnei@ucalgary.ca (N.M.R.M.); mcdonnci@tcd.ie (C.M.); miranda.hambrook@hotmail.com (M.H.)

**Keywords:** cyclic seleninate esters, thiolsulfinates, disulfides, organoselenium redox catalysts, glutathione peroxide mimetics

## Abstract

Cyclic seleninate esters function as mimetics of the antioxidant selenoenzyme glutathione peroxidase. They catalyze the reduction of harmful peroxides with thiols, which are converted to disulfides in the process. The possibility that the seleninate esters could also catalyze the further oxidation of disulfides to thiolsulfinates and other overoxidation products under these conditions was investigated. This has ramifications in potential medicinal applications of seleninate esters because of the possibility of catalyzing the unwanted oxidation of disulfide-containing spectator peptides and proteins. A variety of aryl and alkyl disulfides underwent facile oxidation with hydrogen peroxide in the presence of catalytic benzo-1,2-oxaselenolane *Se*-oxide affording the corresponding thiolsulfinates as the principal products. Unsymmetrical disulfides typically afforded mixtures of regioisomers. Lipoic acid and *N*,*N*′*-*dibenzoylcystine dimethyl ester were oxidized readily under similar conditions. Although isolated yields of the product thiolsulfinates were generally modest, these experiments demonstrate that the method nevertheless has preparative value because of its mild conditions. The results also confirm the possibility that cyclic seleninate esters could catalyze the further undesired oxidation of disulfides *in vivo*.

## 1. Introduction

**Scheme 1:**
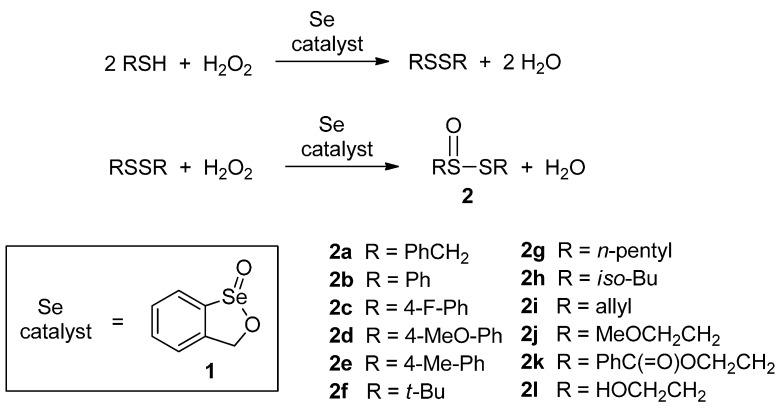
Catalytic reduction of hydrogen peroxide with thiols in the presence of cyclic seleninate ester **1** as catalyst.

**Scheme 2:**
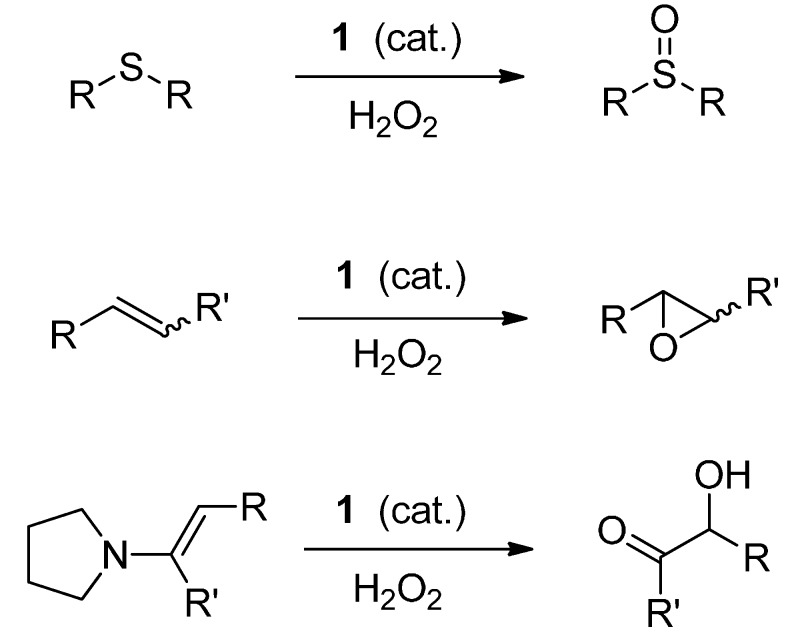
Catalytic oxidation of sulfides, alkenes and enamines with hydrogen peroxide and cyclic seleninate ester **1** as catalyst.

The selenoenzyme family of glutathione peroxidases (GPx) catalyzes the reduction of harmful hydrogen peroxide and lipid hydroperoxides by the stoichiometric reductant glutathione. This process provides protection to living organisms against oxidative stress caused by peroxides and other reactive oxygen species they generate, such as the superoxide radical anion and the hydroxyl radical [1,2,3]. Consequently, the design and synthesis of small-molecule selenium compounds that emulate the function of GPx has proven of interest as a means to suppress oxidative stress that is implicated in a variety of disease states [4,5,6,7,8,9]. Our group [10,11,12,13,14] and that of H.B. Singh [15,16,17] have independently investigated the use of various types of cyclic seleninate esters for this purpose. Thus, seleninate ester **1** and its congeners effectively catalyze the *in vitro* conversion of benzyl thiol to the corresponding disulfide with hydrogen peroxide, as shown in Scheme 1. In the course of this work, we recently observed that in the case of certain related seleninate esters, the concentration of the disulfide reached a maximum at *ca.* 50%–60% completion of the reaction and then began to decrease in the later stages of the process [18]. Such unexpected behaviour suggested that the catalyst might be promoting further oxidation of the initially formed disulfide in this model system. This hypothesis was confirmed by the isolation of thiolsulfinate **2a** (R = benzyl in Scheme 1) and the detection of products of its further decomposition from the late-stage reaction mixtures [18]. In order to gain further insight into this process, which has both biological relevance for these and other GPx mimetics, as well as potential synthetic utility, we embarked on a further study of the oxidation of dibenzyl disulfide with hydrogen peroxide in the presence of **1**, as well as its extension to a variety of other symmetrical and unsymmetrical disulfides. It is also worth noting that **1** is an effective catalyst for the oxidation of sulfides to sulfoxides, alkenes to epoxides and enamines to α-hydroxy ketones (Scheme 2) [19].

Furthermore, it is relevant to note that thiolsulfinates have interesting biological properties. They are produced by various *Allium* or *Brassica* species when chopped or crushed and contribute to the odour and flavour of these plants [20]. Furthermore, some thiolsulfinates have been reported to display antibacterial, antifungal, antiviral and anticancer activity [20,21,22,23,24,25,26,27]. For example, allicin (**2i**) is the compound that is chiefly responsible for the reported antimicrobial activity of garlic. This compound was discovered by Cavallito *et al.* in 1944 [21] and its biogenesis was later shown to proceed by C-S bond cleavage of (*S*)-allylcysteine (*S*)-oxide, catalyzed by the enzyme alliinase and resulting in the formation of allyl sulfenic acid. Condensation of two molecules of the latter with loss of water then affords **2i** [27]. Recently, thiolsulfinates have been shown to function as antioxidants and radical inhibitors [28,29]. 

There are several existing methods for the preparation of thiolsulfinates. The most common ones involve the oxidation of the corresponding disulfides with peracids [21,22,28,30,31,32,33]. Other oxidants that have been employed include singlet oxygen [31,34], dioxiranes [35] and hydrogen peroxide or *tert*-butyl hydroperoxide in the presence of vanadium [36,37], rhenium [38] molybdenum [39] or tungstic acid [40] catalysts, or in protic acid [41] media. Uncatalyzed hydrogen peroxide reacts very slowly with disulfides [42]. Connective methods via the reactions of sulfinyl chlorides [43,44], sulfinic acids [45] or sulfinamides [46] with thiols or tin thiolates [43] provide alternative routes to thiolsulfinates. Several enantioselective variations have also been reported [33,35,36,37,46]. Thiolsulfinates vary greatly in stability and their preparation is often capricious. Exposure to acidic or basic media, or even to silica-gel chromatography, can result in their decomposition. Furthermore, they are prone to overoxidation to thiolsulfonates and other products [30,47,48,49,50,51]. The yields of thiolsulfinates obtained by these methods are therefore highly variable and substrate-dependent. Many of the products described in the older literature are poorly characterized. These considerations, the inexpensive and environmentally benign nature of hydrogen peroxide, as well as our prior success in catalyzing the processes shown in Scheme 1 and Scheme 2 with cyclic seleninate ester **1** prompted us to investigate the oxidation of disulfides further with hydrogen peroxide in the presence of **1**. Finally, additional insight was required into the possibility that cyclic seleninate esters such as **1** could catalyze the unintended oxidation of disulfide bonds in spectator peptides and proteins during applications as biological antioxidants and therapeutic GPx mimetics.

## 2. Results and Discussion

The optimization of the preparation of thiolsulfinate **2a** from dibenzyl disulfide is summarized in Table 1. In the examples in the table, the disulfide and hydrogen peroxide, each at a concentration of 0.1 M, were allowed to react in the indicated solvent at room temperature, in the presence of catalyst **1**. The reaction in chloroform proved relatively slow, achieving less than 50% completion after more than 4 h (entry 1). The use of acetonitrile, a 70:30 mixture of ethyl acetate and methanol or dichloromethane resulted in more rapid reaction rates with improved conversion to the product **2a** and higher isolated yields (entries 2–4, respectively). A decrease in the amount of catalyst from 10 mol % to 5 or 1 mol % reduced the reaction rate drastically (entries 5 and 6). A 95:5 mixture of dichloromethane and methanol with 10 mol % of **1** afforded optimum conditions, with isolation of 64% of the thiolsulfinate (entry 7). In our previous work [19], we had observed that the presence of trifluoroacetic acid (TFA) accelerated the oxidations shown in Scheme 2, while basic conditions suppressed them. The same is evident in the present case, where the inclusion of 20 mol % of TFA accelerated the reaction in dichloromethane-methanol considerably and afforded a comparable yield of the product (entry 8), while the presence of 20 mol % of cesium carbonate inhibited the oxidation completely (entry 9). Since some thiolsulfinates proved more sensitive to acidic conditions than **2a**, we chose the conditions of entry 7 for further applications. A control reaction in the absence of the cyclic seleninate **1** revealed that the uncatalyzed background oxidation of the disulfide with hydrogen peroxide is too slow to contribute significantly to the reaction (entry 10). Finally, with the substrate and conditions indicated in Table 1, only traces of the corresponding thiolsulfonate **3a** (PhCH_2_SSO_2_CH_2_Ph) were detected.

**Table 1:** Optimization of conditions for the synthesis of thiolsulfinate **2a** ^a^. 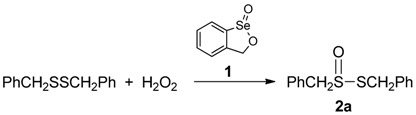

Entry	Solvent	Catalyst 1, mol % (+ Additive)	Time (h)	Ratio of 2a:SM ^b^	Yield ^c^, %
1	CHCl_3_	10	4.25	43:57	15
2	MeCN	10	1.0	85:15	46
3	EtOAc–MeOH (70:30)	10	3.0	85:15	57
4	CH_2_Cl_2_	10	2.0	85:15	53
5	CH_2_Cl_2_	5	4.5	62:38	35
6	CH_2_Cl_2_	1	29	13:87	8
7	CH_2_Cl_2_–MeOH (95:5)	10	3.0	92:8	64
8	CH_2_Cl_2_–MeOH (95:5)	10 (+ 20 mol % TFA)	1.25	^d^	66
9	CH_2_Cl_2_–MeOH (95:5)	10 (+ 20 mol % Cs_2_CO_3_)	6.0	0:100	0
10	CH_2_Cl_2_–MeOH (95:5)	0	24	0:100	0

^a^: Conditions: equimolar amounts of PhCH_2_SSCH_2_Ph and H_2_O_2_ were used at room temperature; ^b^: Ratio determined by ^1^H-NMR integration of the crude material prior to chromatography; SM = starting material; ^c^: Isolated yields of thiolsulfinate **2a** are reported; ^d^: In addition to **2a**, a complex mixture of byproducts was formed.

The conditions of Table 1, entry 7, which also most closely resemble those of our assay for GPx-like activity [10,11,12,13,14], were then applied to a variety of symmetrical and unsymmetrical disulfides (Table 2 and Table 3, respectively). The oxidations of symmetrical diaryl disulfides are provided in entries 2–5 of Table 2, while entry 1 shows the oxidation of the benzyl derivative **2a** for comparison. Oxidation of the electron-rich diaryl disulfide in entry 4 was accompanied by significant thiolsulfonate formation, while the electron-withdrawing *p*-fluoro substituent in entry 3 suppressed reactivity, resulting in a substantial amount of the unreacted disulfide under the usual conditions. The phenyl and 4-fluorophenyl derivatives **2b** and **2c** proved difficult to isolate and purify, resulting in a significant loss of material, although TLC indicated their relatively clean initial conversion from the corresponding disulfides. In general, the formation of the thiolsulfinates was accompanied by small amounts of thiolsulfonates **3**, along with minor quantities of the starting disulfides. The mixtures were separated by flash chromatography on silica gel, which was carried out rapidly because some of the thiolsulfinates were prone to decomposition during the process. The chromatographic separation of **2d** and **3d** (entry 4) could not be achieved and the yield is reported for the unseparated mixture. The conversions of the dialkyl disulfides in entries 6–9 to the corresponding thiolsulfinates **2f**–**2i** proceeded smoothly, even in the case of the hindered *t*-butyl product **2f**. The ether- and ester-substituted products **2j** and **2k**, respectively, showed no sign of overoxidation to **3j** and **3k**, while the 2-hydroxyethyl disulfide in entry 12 failed to undergo any significant oxidation under the usual conditions. Again, substantial losses of the aliphatic products occurred during separation and purification, resulting in relatively modest isolated yields, especially in the case of allicin (**2i**).

**Table 2:** Oxidation of symmetrical disulfides to thiolsulfinates **2** ^a^. 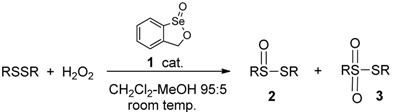

Entry	R	Time (h)	2:3:SM ^b^	Thiolsulfinate	Yield, % ^c^
1	PhCH_2_	3	92:trace:8	2a	64
2	Ph	4	nd ^d^	2b	23
3	4-F-Ph	3	48:trace:52 ^e^	2c	38
4	4-MeO-Ph	4	59:35:6	2d	90 ^f^
5	4-Me-Ph	5	92:trace:8	2e	55
6	*t*-Bu	2.5	84:2:14	2f	41
7	*n*-Pentyl	3	93:trace:7	2g	54
8	*iso-*Bu	3	88:8:4	2h	56
9	Allyl	3	nd	2i	31
10	MeOCH_2_CH_2_	6	80:0:20	2j	59
11	PhC(=O)OCH_2_CH_2_	6	92:0:8	2k	50
12	HOCH_2_CH_2_	4	NR	2l	NR

^a^: Conditions: equimolar amounts of disulfide and H_2_O_2_ along with 10 mol % of **1** in dichloromethane-methanol (95:5) were used at room temperature; ^b^: Ratio of crude product mixture determined by integration of ^1^H-NMR signals, except where otherwise indicated; SM = starting material; ^c^: Isolated yields of thiolsulfinates are reported; NR = no reaction was observed; ^d^: Ratio could not be determined due to absence or overlap of suitable NMR signals for integration; ^e^: Ratio was determined by integration of ^19^F-NMR signals; ^f^: Yield is reported for the unseparated mixture of **2d** and **3d**.

**Table 3:** Oxidation of unsymmetrical disulfides to thiolsulfinates **4** and **5** ^a^. 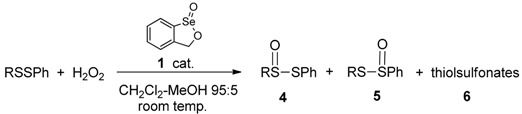

Entry	R	Time (h)	4:5:6:SM ^b^	Thiolsulfinates ^c^	Yield, %^ d^
1	4-MeO-Ph	3	33:33:33:0	4a + 5a	43
2	*t*-Bu	4	67:12:0:21	4b + 5b	63
3	*n*-C_6_H_13_	3.5	68:8:8:16	4c ^e^	47

^a^: Conditions: equimolar amounts of disulfide and H_2_O_2_ along with 10 mol % of **1** in dichloromethane-methanol (95:5) were used at room temperature; ^b^: Ratio in crude product mixture determined by integration of ^1^H-NMR signals; SM = starting material; ^c^: Unseparated mixtures of regioisomers **4** and **5** were obtained; ^d^: Isolated yields of thiolsulfinates are reported; ^e^: Product **4c** was isolated as a pure regioisomer in 47% yield; products **5c** and **6c** could not be separated and their combined yield was 16%.

**Scheme 3:**
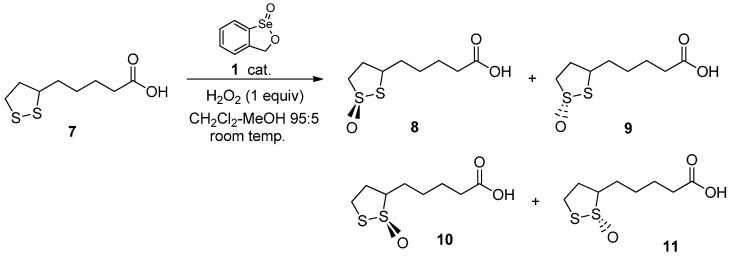
Oxidation of (±)-lipoic acid with hydrogen peroxide and catalyst **1**.

The unsymmetrical disulfides shown in Table 3 were obtained by a literature method [52]. Their oxidations were intrinsically more complex because inseparable regioisomers, as well as overoxidation products, were possible. The aryl-substituted product in entry 1 was produced as a mixture of equal amounts of the two regioisomers **4a** and **5a**. On the other hand, the phenyl alkyl-substituted disulfides in entries 2 and 3 afforded predominantly the regioisomers **4**, where oxidation occurred proximally to the alkyl substituent, even in the case of the hindered *t*-butyl derivative **4b**. The greater reactivity of the more electron-rich alkyl-substituted sulfur atom is expected, and has been observed previously with other electrophilic oxidants [30]. The thiolsulfinates in entries 1 and 3 were again accompanied by small amounts of regioisomeric mixtures of thiolsulfonates. The similar oxidation of racemic lipoic acid (**7**) afforded roughly equal amounts of four inseparable products **8**–**11**. Based on the NMR spectra of the mixture, we conclude that it consists of the two regioisomers, each formed as mixtures of two diastereomers as a result of the chiral nature of the thiolsulfinate moiety (Scheme 3).

**Scheme 4:**
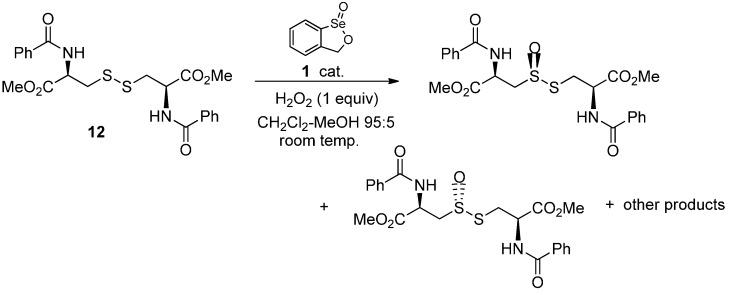
Oxidation of a cystine derivative with hydrogen peroxide and catalyst **1**.

Finally, we investigated the oxidation of (*L*)-*N*,*N*′-dibenzoylcystine dimethyl ester (**12**) under the usual conditions, catalyzed by seleninate ester **1**. The complete consumption of the starting material was observed after 6 h, resulting in a complex mixture of products that could not be separated. However, the NMR and mass spectra of the mixture were consistent with the formation of the stereoisomers of the corresponding thiolsulfinate, in addition to unidentified material (Scheme 4). This suggests that cyclic seleninate ester **1** and its congeners could be capable of oxidizing disulfide linkages in native proteins and peptides in the presence of peroxides when employed as GPx mimetics *in vivo*.

**Scheme 5:**
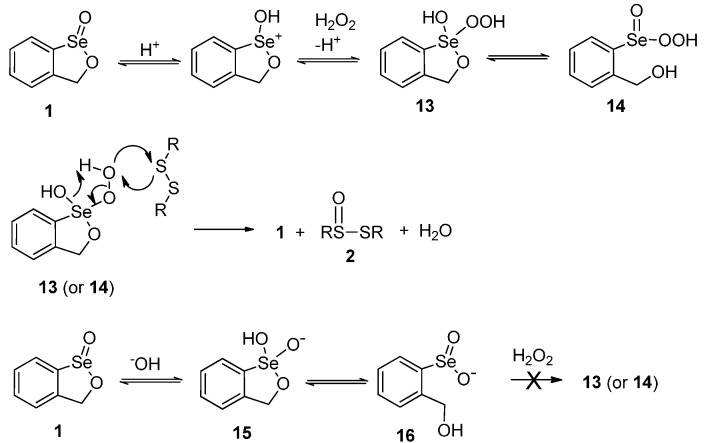
Mechanism for the oxidation of disulfides with hydrogen peroxide and catalyst **1**.

While no direct mechanistic studies were carried out on the present oxidations of disulfides to thiolsulfinates, the process very likely proceeds via a similar pathway to that described previously for the oxidations shown in Scheme 2 [19]. This involves the initial formation of a peroxyseleninic acid **14** or peroxyselenurane **13** from **1** and hydrogen peroxide, which is enhanced by prior protonation of **1** with an acid catalyst. This is followed by oxygen transfer to the most electron-rich sulfur atom of the disulfide, as shown in Scheme 5. Failure of the reaction under basic conditions is attributed to the formation of anions **15** or **16**, which are unable to react further with hydrogen peroxide to generate the active oxidant intermediates **13** or **14**.

## 3. Experimental Section

### 3.1. General Information

Symmetrical disulfides were obtained from commercial sources, while unsymmetrical disulfides were prepared by thiolysis of *N*-(phenylthio)phthalimide via the method of Harpp *et al.* [52] except that toluene instead of carbon tetrachloride was employed as the solvent. The preparation of cyclic seleninate ester **1** was carried out by the previously described procedure [11,13]. Hydrogen peroxide (*ca.* 30%) was titrated prior to use [53]. ^1^H-NMR spectra were recorded at 400 MHz, while ^13^C- and ^77^Se-NMR spectra were obtained at 101 and 76 MHz, respectively. Chemical shifts of ^77^Se-NMR spectra were obtained with diphenyl diselenide in CDCl_3_ (463.0 ppm) [54] as the standard, relative to dimethyl selenide (0.0 ppm), while C_6_F_6_ (−164.9 ppm) in CDCl_3_, relative to CFCl_3_ (0.0 ppm), was the standard for ^19^F-NMR spectra [55]. High resolution mass spectra were obtained using a time of flight (TOF) analyzer with electron impact (EI) ionization or a quadrupole TOF analyzer with electrospray ionization (ESI).

### 3.2. Typical Procedure: Preparation S-Benzyl Phenylmethanesulfinothioate *(**2a**)*

Dibenzyl disulfide (245 mg, 1.00 mmol) and cyclic seleninate ester **1** (20 mg, 0.10 mmol) were dissolved in 10 mL of dichloromethane at room temperature with stirring. Hydrogen peroxide (127 μL, 26.4% *w*/*v*, 1.00 mmol) was added to the solution. The mixture was stirred at room temperature and progress was monitored by TLC. After 3 h, the reaction was diluted with dichloromethane, washed with brine and dried with Na_2_SO_4_. The product was purified by flash chromatography on silica gel (hexane-ethyl acetate, 10:1) to afford 139 mg (53%) of **2a**. The product had properties identical to those reported previously [18]. When the reaction was repeated in dichloromethane-methanol (95:5), a slightly higher yield of 64% was obtained. The other thiolsulfinates in Table 2 and Table 3 were prepared similarly, with any changes to the above conditions as noted in the Tables. Characterization data for the other products follows.

### 3.3. S-Phenyl Benzenesulfinothioate *(**2b**)*

Pale yellow powder (23%; mixture of **2b** (major) [43] and **3b** (minor); IR (film) 1333, 1152, 1100, 1052, 743 cm^−1^; ^1^H-NMR (400 MHz, CDCl_3_) δ 7.66 (dd, *J* = 6.6, 3.0 Hz, 2 H), 7.55–7.33 (m, 8 H); ^13^C-NMR (101 MHz, CDCl_3_) δ 135.3, 131.5, 130.3, 129.2, 128.9, 124.3, 114.4 (signals from thiolsulfonate **3b**: δ 144.0, 136.6, 129.4, 129.3; 128.7, 127.5; HRMS (ESI-TOF) *m/z* [M + Na]^+^ calcd for C_12_H_10_NaOS_2_: 257.0065; found: 257.0065.

### 3.4. S-4-Fluorophenyl 4-Fluorobenzenesulfinothioate *(**2c**)*

White solid (38%) [49,50]; mp 73–75 °C, lit. [49] mp 75.5–76.5 °C; IR (film) 1395, 1233, 1109, 1081, 833 cm^−1^; ^1^H-NMR (400 MHz, CDCl_3_) δ 7.63–7.56 (m, 2 H), 7.46–7.40 (m, 2 H), 7.17 (t, *J* = 8.6 Hz, 2 H), 7.07 (t, *J* = 8.6 Hz, 2 H); ^13^C-NMR (101 MHz, CDCl_3_) δ 164.5 (d, *J* = 253.1 Hz, CF), 164.4 (d, *J* = 252.2 Hz, CF), 139.3 (d, *J* = 3.1 Hz), 137.8 (d, *J* = 9.0 Hz), 126.6 (d, *J* = 9.2 Hz), 123.8 (d, *J* = 3.5 Hz), 116.5 (d, *J* = 21.9 Hz), 116.3 (d, *J* = 22.8 Hz); ^19^F-NMR (376 MHz, CDCl_3_) δ −107.1, −109.1; HRMS (ESI-TOF) *m/z* [M + H]^+^ calcd for C_12_H_9_F_2_OS_2_: 271.0057; found 271.0053; *m/z* [M + Na]^+^ calcd for C_12_H_8_F_2_NaOS_2_: 292.9877; found: 292.9874.

### 3.5. S-4-Methoxyphenyl 4-Methoxybenzenesulfinothioate *(**2d**)*

The product was isolated as an inseparable mixture of the thiolsulfinate **2d** [39] and thiolsulfonate **3d** as a yellow oil (90% total; 63:37). IR (film) 1323, 1319, 1167, 1133, 1100, 829 cm^−1^; ^1^H-NMR (400 MHz, CDCl_3_) signals from **2d**: δ 7.58 (crude d, *J* = 8.9 Hz, 2 H), 7.44 (crude d, *J* = 8.8 Hz, 2 H), 7.01 (crude d, *J* = 8.8 Hz, 2 H), 6.92 (crude d, *J* = 8.8 Hz, 2 H), 3.88 (m, 3 H), 3.86 (s, 3 H); signals from **3d**: δ 7.54 (crude d, *J* = 8.9 Hz, 2 H), 7.30 (crude d, *J* = 8.9 Hz, 2 H), 6.94–6.85 (m, 4 H), 3.89 (s, 3 H), 3.85 (s, 3 H); ^13^C-NMR (101 MHz, CDCl_3_) δ (both **2d** and **3d**) 163.6, 162.3, 161.8, 138.5. 137.6, 135.3, 135.1, 130.0, 126.3, 120.1, 119.1, 115.0, 114.9, 114.6, 114.5, 113.9, 55.8, 55.7, 55.59, 55.57; HRMS of **2d** (ESI-TOF) *m/z* [M + H]^+^ calcd for C_14_H_15_O_3_S_2_: 295.0457; found: 295.0448; HRMS of **3d** (ESI-TOF) *m/z* [M + H]^+^ calcd for C_14_H_15_O_4_S_2_: 311.0406; found: 311.0402.

### 3.6. S-p-Tolyl 4-Methylbenzenesulfinothioate *(**2e**)*

White powder (55%); mp 85–86 °C, lit [56]. mp 87.5 °C; IR (film) 1634, 1424, 1087, 988, 729 cm^−1^; ^1^H-NMR (400 MHz, CDCl_3_) δ 7.57 (d, *J* = 8.3 Hz, 2 H), 7.44 (d, *J* = 8.2 Hz, 2 H), 7.30 (d, *J* = 8.0 Hz, 2 H), 7.19 (d, *J* = 7.9 Hz, 2 H), 2.42 (s, 3 H), 2.39 (s, 3 H); ^13^C-NMR (101 MHz, CDCl_3_) δ 142.1, 141.1, 140.8, 135.4, 130.1, 129.6, 126.3, 124.4, 21.5, 21.4; HRMS (ESI-TOF) *m/z* [M + H]^+^ calcd for C_14_H_15_OS_2_: 263.0562; found: 263.0559.

### 3.7. S-tert-Butyl 2-Methylpropane-2-sulfinothioate *(**2f**)*

Golden yellow oil [34,57] (41%); IR (film) 1367, 1167, 1090, 514 cm^−1^; ^1^H-NMR (400 MHz, CDCl_3_) δ 1.53 (s, 9 H), 1.35 (s, 9 H); ^13^C-NMR (101 MHz, CDCl_3_) δ 59.6, 48.8, 32.5, 24.4 ppm; HRMS (EI-TOF) *m/z* [M]^+^ calcd for C_8_H_18_OS_2_: 194.0799; found: 194.0803.

### 3.8. S-n-Pentyl Pentane-1-sulfinothioate *(**2g**)*

Pale yellow oil [22] (54%); IR( film) 1085, 728 cm^−1^; ^1^H-NMR (400 MHz, CDCl_3_) δ 3.16–3.00 (m, 4 H), 1.83–1.75 (m, 4 H), 1.41–1.32 (m, 8 H), 0.91 (t, *J* = 7.2 Hz, 3 H), 0.88 (t, *J* = 7.1 Hz, 3 H); ^13^C-NMR (101 MHz, CDCl_3_) δ 56.3, 32.9, 30.7, 30.5, 23.2, 22.3, 22.1, 13.9, 13.8; HRMS (EI-TOF) *m/z* [M]^+^ calcd for C_10_H_22_OS_2_: 222.1112; found: 222.1118.

### 3.9. S-Isobutyl 2-Methylpropane-1-sulfinothioate *(**2h**)*

Pale yellow oil [58] (56%); IR (film) 1390, 1085, 1057, 485 cm^−1^; ^1^H-NMR (400 MHz, CDCl_3_) δ 3.10 (dd, *J* = 12.9, 5.8 Hz, 1 H), 3.05 (dd, *J* = 13.4, 6.7 Hz, 1 H), 2.97 (dd, *J* = 13.4, 6.7 Hz, 1 H), 2.89 (dd, *J* = 12.9, 8.5 Hz, 1 H), 2.26–2.20 (m, 1 H), 2.01–1.98 (m, 1 H), 1.07 (d, *J* = 6.7 Hz, 6 H), 1.01 (d, *J* = 6.6 Hz, 3 H), 1.00 (d, *J* = 6.6 Hz, 3 H); ^13^C-NMR (101 MHz, CDCl_3_) δ 65.1, 41.6, 29.7, 24.9, 22.5, 21.64, 21.61, 21.5; HRMS (EI-TOF) *m/z* [M]^+^ calcd for C_8_H_18_OS_2_: 194.0799; found: 194.0798.

### 3.10. S-Allyl Prop-2-ene-1-sulfinothioate allicin *(**2i**)*

Pale yellow oil [21,22] (31%); IR (film) 1264, 1094, 1067, 808, 739 cm^−1^; ^1^H-NMR (400 MHz, CDCl_3_) δ 5.99–5.86 (m, 2 H), 5.46–5.16 (m, 4 H), 3.87–3.69 (m, 4 H); ^13^C-NMR (101 MHz, CDCl_3_) δ 133.1, 126.1, 124.2, 119.3, 60.1, 35.2; HRMS (EI-TOF) *m/z* [M]^+^ calcd for C_6_H_10_OS_2_: 162.0173; found: 162.0165.

### 3.11. S-2-Methoxyethyl 2-Methoxyethanesulfinothioate *(**2j**)*

Yellow oil [59] (59%); IR (film) 1376, 1119, 1081, 952 cm^−1^; ^1^H-NMR (400 MHz, CDCl_3_) δ 3.89–3.77 (m, 2 H), 3.76–3.66 (m, 2 H), 3.40 (s, 3 H), 3.39 (s, 3 H), 3.36–3.26 (m, 4 H); ^13^C-NMR (101 MHz, CDCl_3_) δ 71.8, 65.4, 59.0, 58.8, 56.4, 32.7; HRMS (ESI-TOF) *m/z* [M + H]^+^ calcd for C_6_H_15_O_3_S_2_: 199.0457; found: 199.0454.

### 3.12. S-2-Benzoyloxyethyl 2-Benzoylethanesulfinothioate *(**2k**)*

White solid (50%); mp 57–58 °C; IR (film) 1729, 1262, 1109, 1067, 1024 cm^−1^; ^1^H-NMR (400 MHz, CDCl_3_) δ 8.07–8.02 (m, 4 H), 7.61–7.54 (m, 2 H), 7.47–7.40 (m, 4 H), 4.84 (dt, *J* = 12.3, 5.3 Hz, 1 H), 4.75 (ddd, *J* = 12.3, 6.9, 5.3 Hz, 1 H), 4.70–4.58 (m, 2 H), 3.65–3.55 (m, 3 H), 3.51 (dt, *J* = 14.5, 6.5 Hz, 1 H); ^13^C-NMR (101 MHz, CDCl_3_) δ 166.1, 166.0, 133.4, 133.2, 129.74, 129.68, 129.5, 129.3, 128.5, 128.4, 63.8, 57.8, 55.2, 31.9; HRMS (ESI-TOF) *m/z* [M + H]^+^ calcd for C_18_H_18_O_5_S_2_: 379.0668; found: 379.0662.

### 3.13. S-Phenyl 4-Methoxybenzenesulfinothioate (**4a**) and S-4-Methoxyphenyl Benzenesulfino-thioate *(**5a**)*

The product was isolated as a white solid (43% of a *ca.* 50:50 mixture of the two regioisomers [60,61]); IR (film) 1257, 1171, 1095, 1057, 823 cm^−1^; ^1^H-NMR (400 MHz, CDCl_3_) signals for both isomers: δ 7.70–7.30 (m, 7 H), 7.00 (crude d, *J* = 8.8 Hz, 1H), 6.89 (crude d, *J* = 8.8 Hz, 1H), 3.87 and 3.84 (s, 3 H for each isomer); ^13^C-NMR (101 MHz, CDCl_3_) δ 162.3, 161.7, 137.5, 135.2, 131.3, 130.1, 128.8, 126.1, 124.3, 114.8, 114.4, 55.5, 55.4; HRMS (ESI-TOF) of both regioisomers *m/z* [M + H]^+^ calcd for C_13_H_13_O_2_S_2_: 265.0351; found: 265.0348.

### 3.14. S-Phenyl 2-Methylpropane-2-sulfinothioate *(**4b**) [24]* and S-tert-Butyl Benzenesulfinothioate *(**5b**)*

The product [43] was isolated as a yellow oil (63% of a 85:15 mixture of regioisomers); IR (film) 1371, 1361, 1171, 1167, 1095, 1085, 1057, 1014, 757, 752 cm^−1^; NMR signals of the major regioisomer **4c**: ^1^H-NMR (400 MHz, CDCl_3_) δ 7.64 (dd, *J* = 7.5, 2.1 Hz, 2 H), 7.42–7.37 (m, 3 H), 1.47 (s, 9 H); ^13^C-NMR (101 MHz, CDCl_3_) δ 135.0, 129.9, 129.6, 129.4, 60.3, 24.2; NMR signals of the minor regioisomer **5c**: ^1^H-NMR (400 MHz, CDCl_3_) δ 7.73 (dd, *J* = 8.2, 1.5 Hz, 2H), 7.53–7.45 (m, 3 H), 1.65 (s, 9 H); ^13^C-NMR (101 MHz, CDCl_3_) δ 32.2; HRMS (ESI-TOF) of both regioisomers: *m/z* [M + H]^+^ exact mass calcd for C_10_H_15_OS_2_: 215.0559; found: 215.0556.

### 3.15. S-Phenyl Hexane-1-sulfinothioate *(**4c**)*

Yellow oil (47%); IR (film) 1090, 747 cm^−1^; ^1^H-NMR (400 MHz, CDCl_3_) δ 7.63 (dd, *J* = 7.9, 1.8 Hz, 2H), 7.47–7.38 (m, 3H), 3.13 (t, *J* = 7.7 Hz, 2H), 1.86 (ddd, *J* = 14.7, 7.5, 2.9 Hz, 2H), 1.47 (ddd, *J* = 8.9, 4.1, 1.9 Hz, 2H), 1.34 (dq, *J* = 7.3, 3.7 Hz, 4H), 1.05–0.86 (m, 3H); ^13^C-NMR (101 MHz, CDCl_3_) δ 135.1, 130.1, 129.4, 129.3, 56.1, 31.3, 28.3, 23.4, 22.3, 13.9; HRMS (ESI-TOF) *m/z* [M + H]^+^ calcd for C_12_H_19_OS_2_: 243.0872; found: 243.0871.

### 3.16. S-Hexyl Benzenesulfinothioate *(**5d**)* and S-Hexyl Benzenesulfonothioate *(**6d**)*

The product was isolated as a yellow oil (16% of an 1:1 mixture of the thiolsulfinate **5d** and thiolsulfonate **6d**); IR (film) 1324, 1133, 1095, 1062, 752 cm^−1^; ^1^H-NMR (400 MHz, CDCl_3_) δ 7.74 (dd, *J* = 7.9, 1.7 Hz, 2H), 7.71–7.67 (m, 2H), 7.57–7.43 (m, 6H), 3.29–3.14 (m, 2H), 3.08 (dt, *J* = 13.4, 7.4 Hz, 2H), 1.97–1.85 (m, 2H), 1.79 (pd, *J* = 7.4, 2.9 Hz, 2H), 1.40 (dq, *J* = 12.3, 7.3 Hz, 4H), 1.35–1.26 (m, 10H), 0.89 (td, *J* = 7.0, 2.6 Hz, 6H); ^13^C-NMR (101 MHz, CDCl_3_) δ 136.3, 131.4, 131.3, 129.7, 129.0, 128.0, 126.9, 124.3, 59.5, 33.29, 31.2, 31.1, 30.5, 28.3, 27.6, 23.4, 22.4, 22.2, 13.9, 13.9; HRMS (ESI-TOF) of **5d**: *m/z* [M + H]^+^ calcd for C_12_H_19_OS_2_: 243.0872; found: 243.087; HRMS (ESI-TOF) of **6d**: *m/z* [M + NH_4_]^+^ exact mass calcd for C_12_H_22_NO_2_S_2_: 276.1086; found: 276.10.

### 3.17. Oxidation of (±)-Lipoic Acid *(**7**)*

The oxidation was carried out by the usual procedure. The product was isolated as a clear, colourless oil in quantitative yield, but in *ca.* 90% purity, as attempts to remove residual catalyst **1** failed. The 400 MHz ^1^H-NMR spectrum was extremely complex, but ^13^C-NMR data (see Supporting Information) matched those reported by Müller *et al.* [62] for a similar mixture of all four stereo- and regioisomers. IR (film) 1367, 1195, 1086, 1024 cm^−1^; HRMS (ESI-TOF) *m/z* [M + NH_4_]^+^ calcd for C_8_H_18_NO_3_S_2_: 240.0723; found: 240.0725.

### 3.18. Oxidation of (L)-N,N′-Dibenzoylcystine Dimethyl Ester *(**12**)*

The oxidation was carried out by the usual procedure. The product was a mixture of products, but appears to consist primarily of the two corresponding thiolsulfinate isomers. The methine signal of the starting material at 5.1 ppm was replaced by four new signals between 5.0 and 5.3 ppm, while four new methyl singlets were apparent. The original disulfide methylene signal at 3.4 ppm was absent and a complex multiplet overlapping with the methyl peaks at 3.7–4.0 ppm was observed instead (see Supplementary Materials). HRMS (ESI-TOF) *m/z* [M + H]^+^ calcd for C_22_H_25_N_2_O_7_S_2_: 493.1098; found: 493.1083.

## 4. Conclusions

In conclusion, cyclic seleninate ester **1** is an effective catalyst for the oxidation of disulfides to thiolsulfinates with hydrogen peroxide, even in the absence of acidic cocatalysts. In most cases, overoxidation to thiolsulfonates was largely suppressed by the use of only one equivalent of the peroxide. Regioselectivity was poor or nonexistent with unsymmetrical diaryl disulfides and oxidation at the alkyl-substituted sulfur atom was favoured in aryl alkyl derivatives. Product losses were significant during the isolation and purification of the products. The facile oxidation of cystine analogue **12** under these conditions indicates that damage to spectator disulfide-containing proteins and peptides is possible during the use of **1** or its congeners as GPx mimetics or in other biological applications.

## Supplementary Materials

Supplementary materials can be accessed at: http://www.mdpi.com/1420-3049/20/06/10748/s1.
