# Supplementary material for: Oxidation of Disulfides to Thiolsulfinates with Hydrogen Peroxide and a Cyclic Seleninate Ester Catalyst
Source: Molecules. 2015 Jun 11;20(6):10748–62. doi: 10.3390/molecules200610748 (PMC6272456; doi:10.3390/molecules200610748)
Supplement: Supplementary file 1 [file molecules-20-10748-s001.pdf]

Supplementary Materials

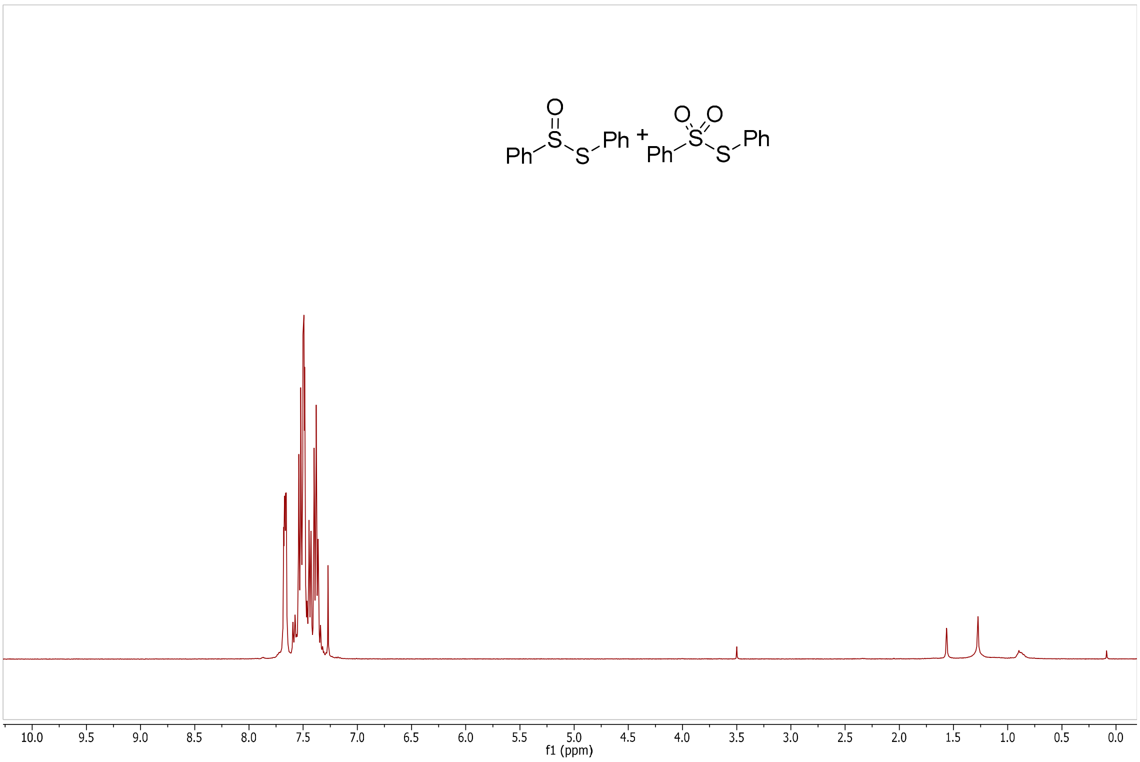

Figure S1. ^{1}H-NMR spectrum of thiolsulfinate **2b** and thiolsulfonate **3b** ($CDCl_3$).

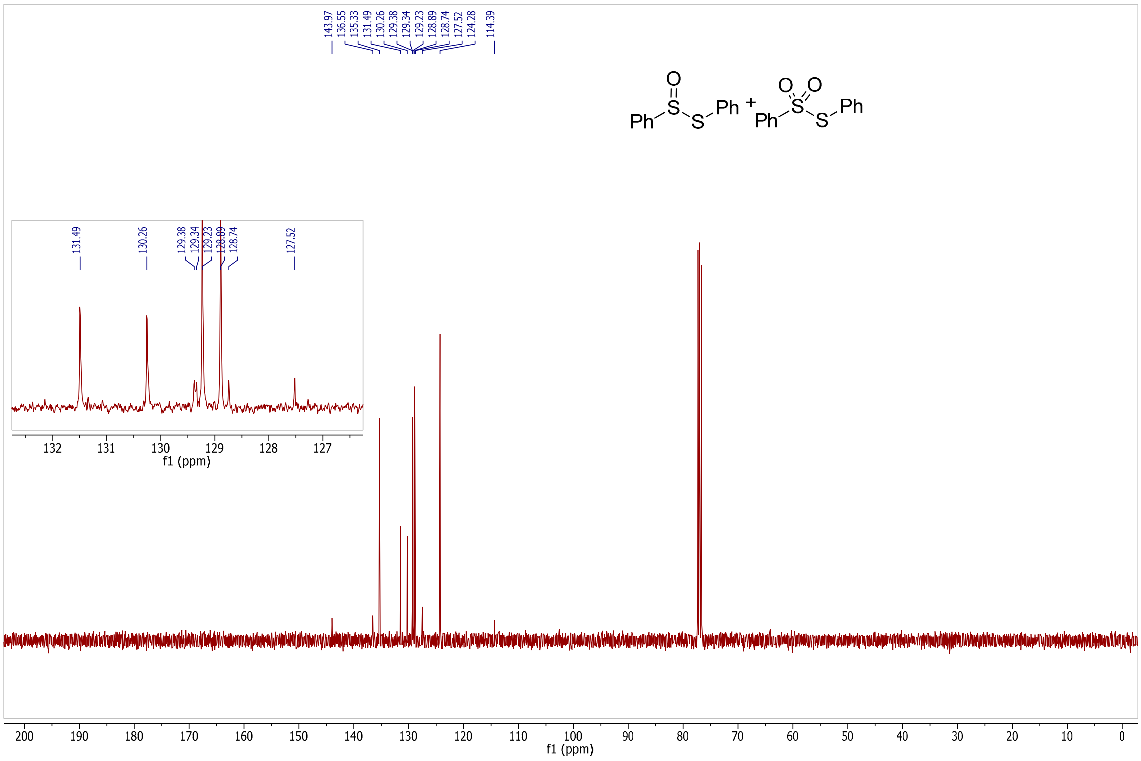

Figure S2. ^{13}C-NMR spectrum of thiolsulfinate **2b** and thiolsulfonate **3b** ($CDCl_3$).

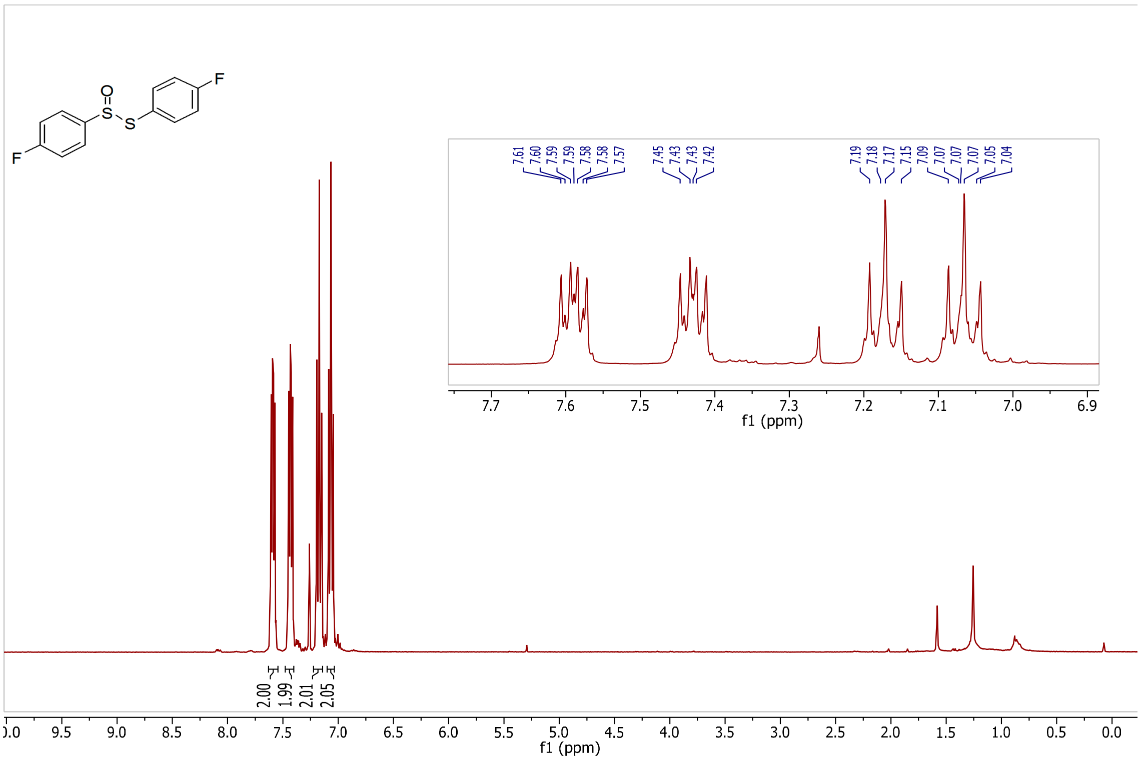

Figure S3. ^{1}H-NMR spectrum of thiolsulfinate **2c** ($CDCl_3$).

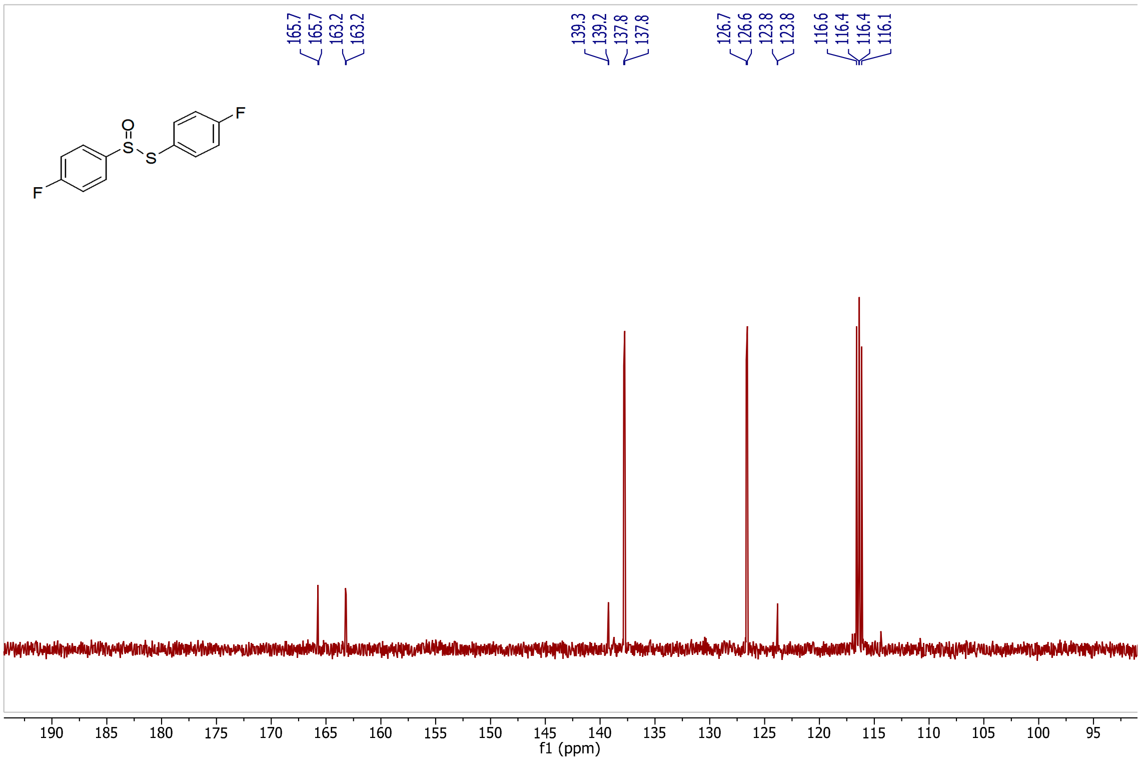

Figure S4. ^{13}C-NMR spectrum of thiolsulfinate **2c** ($CDCl_3$).

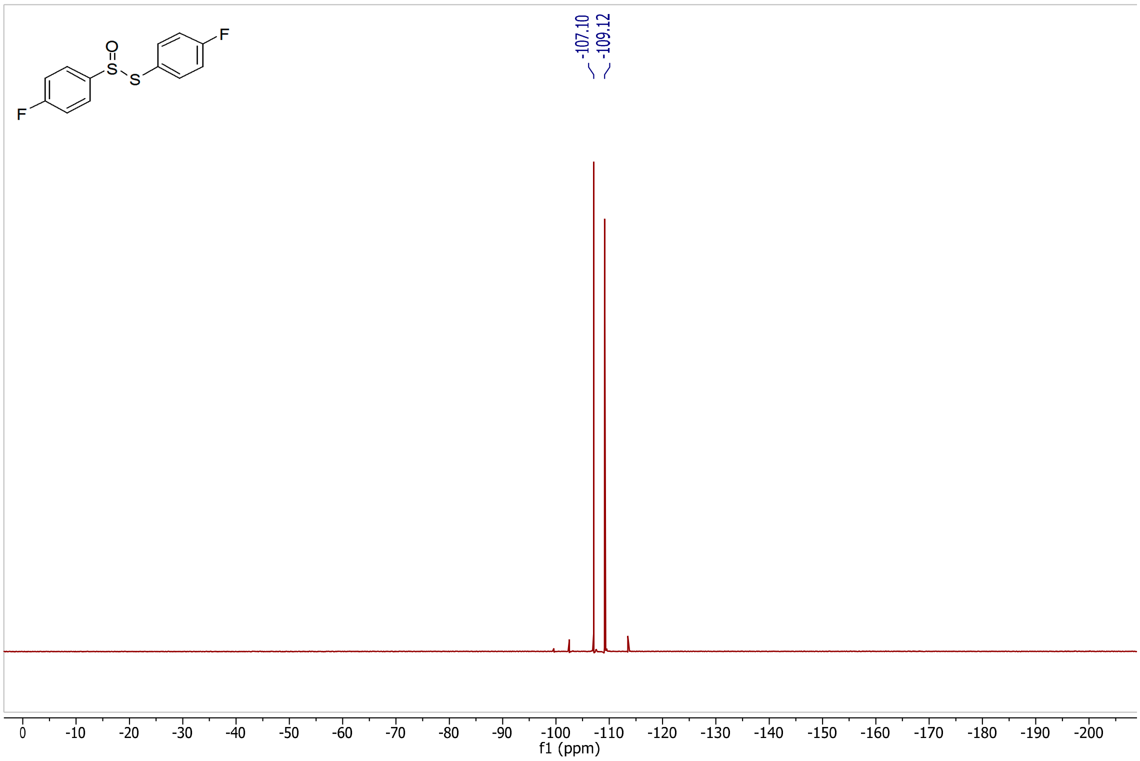

Figure S5. ^{19}F-NMR spectrum of thiolsulfinate **2c** ($CDCl_3$).

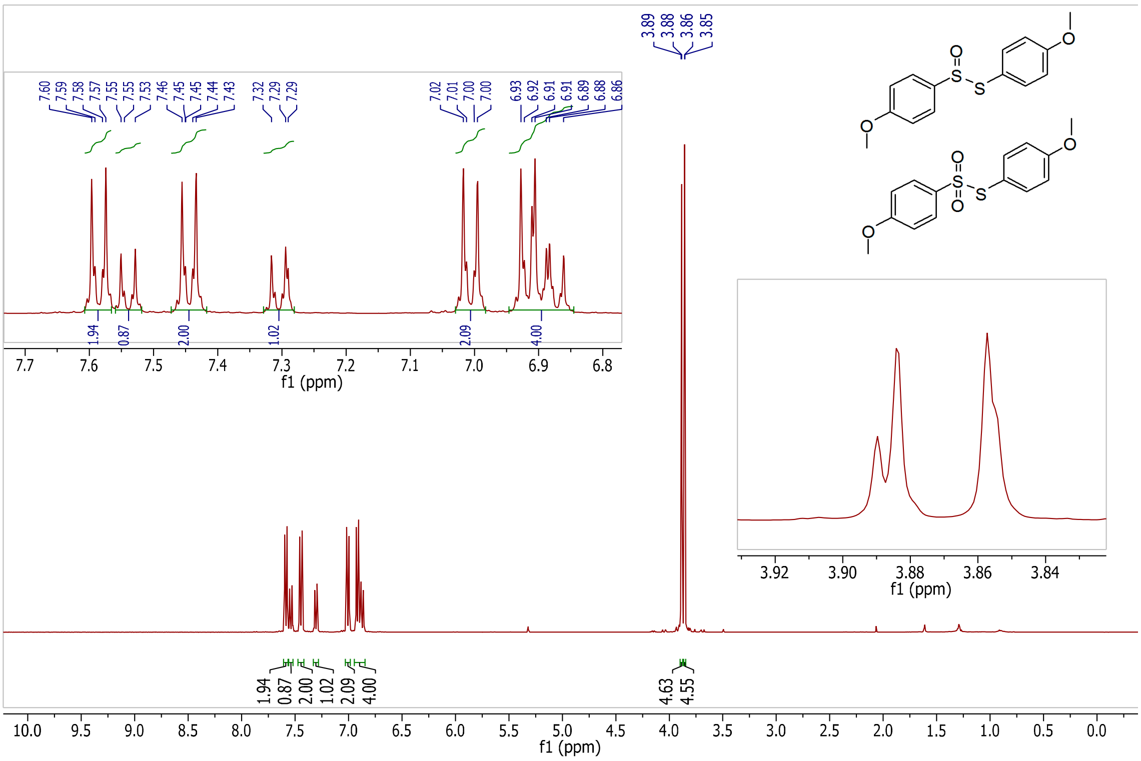

Figure S6. ^{1}H-NMR spectrum of thiolsulfinate **2d** and thiolsulfonate **3d** ($CDCl_3$).

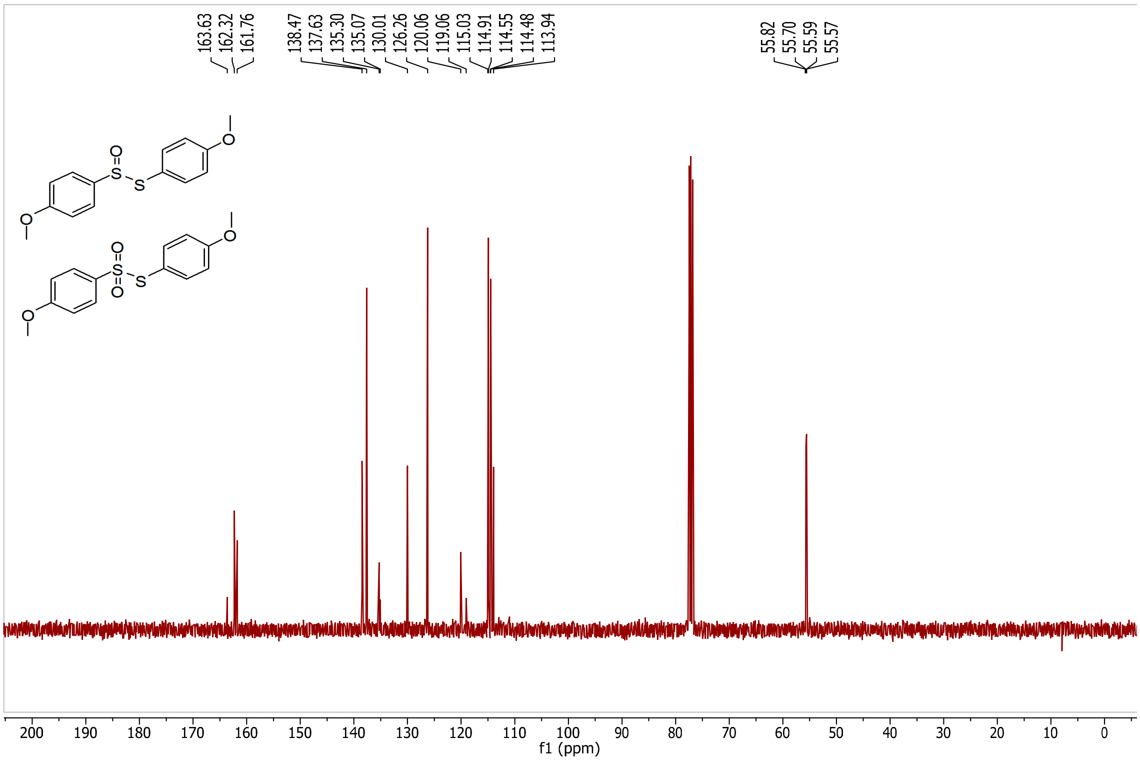

Figure S7. ^{13}C-NMR spectrum of thiolsulfinate **2d** and thiolsulfonate **3d** ($CDCl_3$).

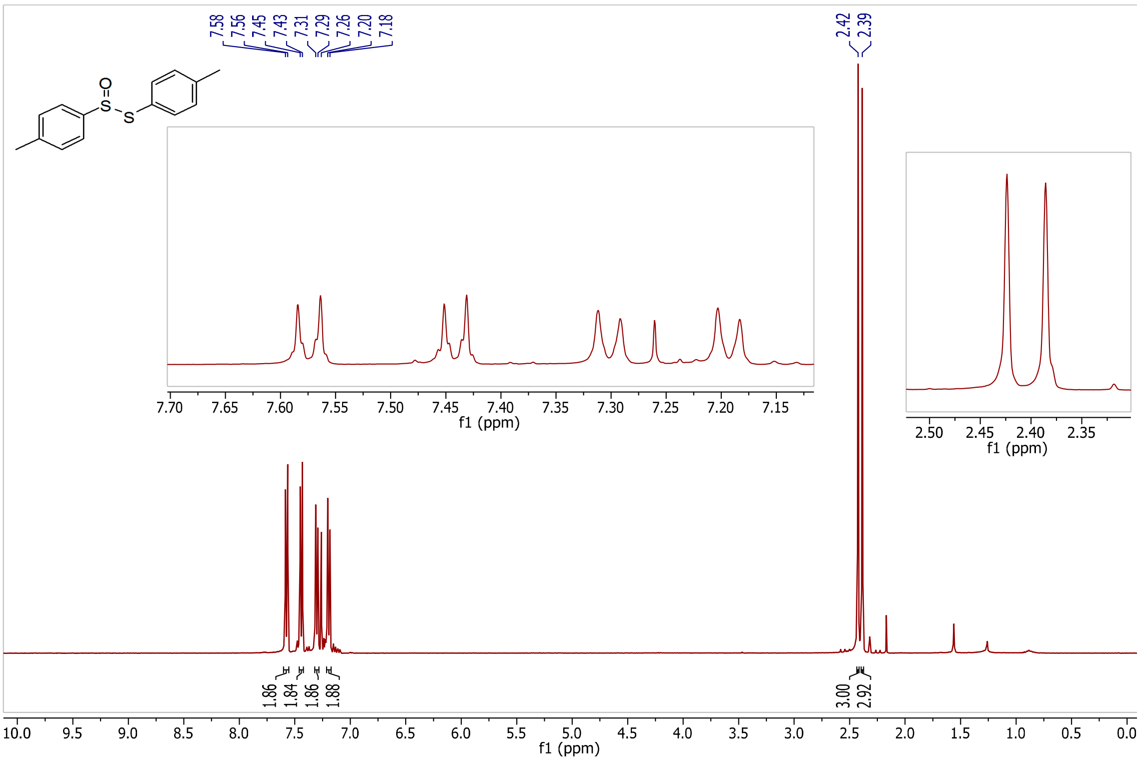

Figure S8. ^{1}H-NMR spectrum of thiolsulfinate **2e** ($CDCl_3$).

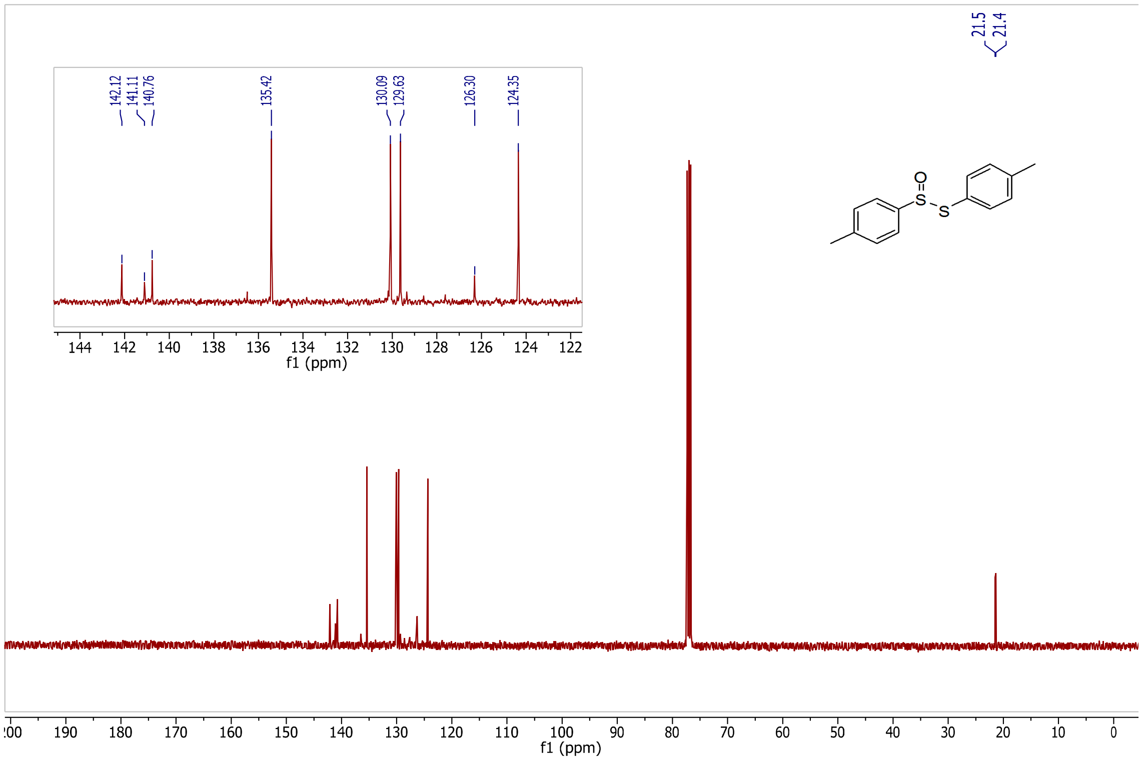

Figure S9. ^{13}C-NMR spectrum of thiolsulfinate **2e** ($CDCl_3$).

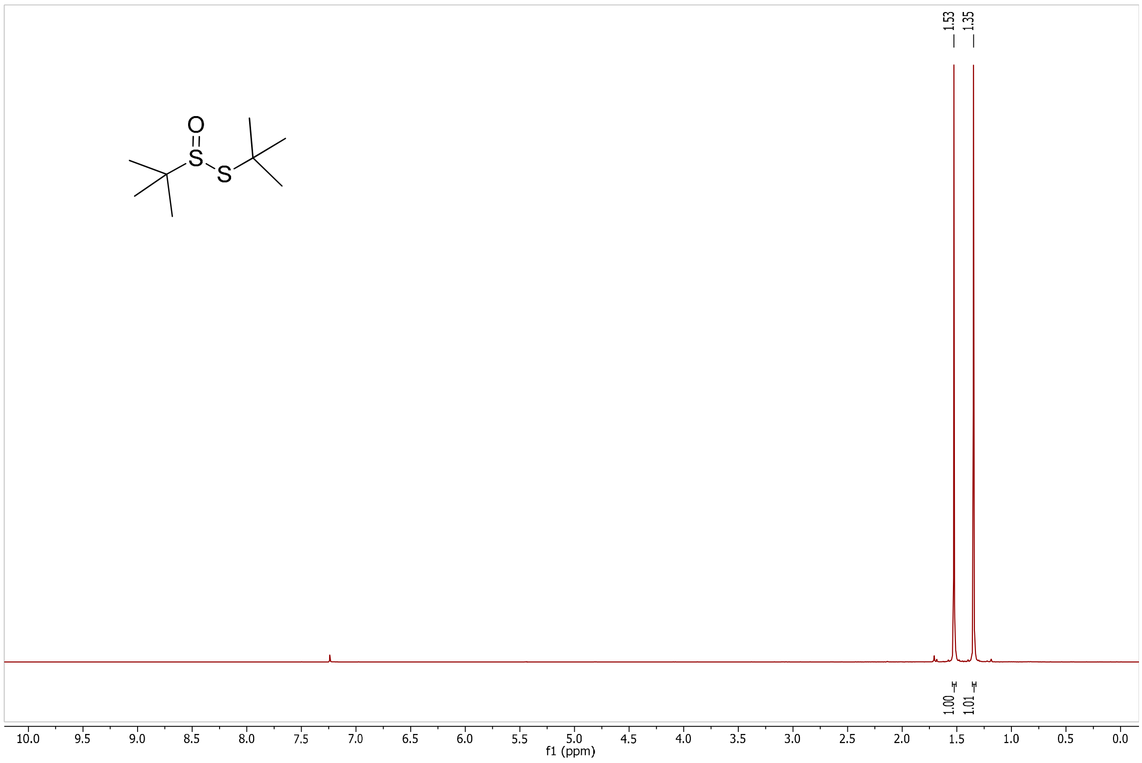

Figure S10. ^{1}H-NMR spectrum of thiolsulfinate **2f** ($CDCl_3$).

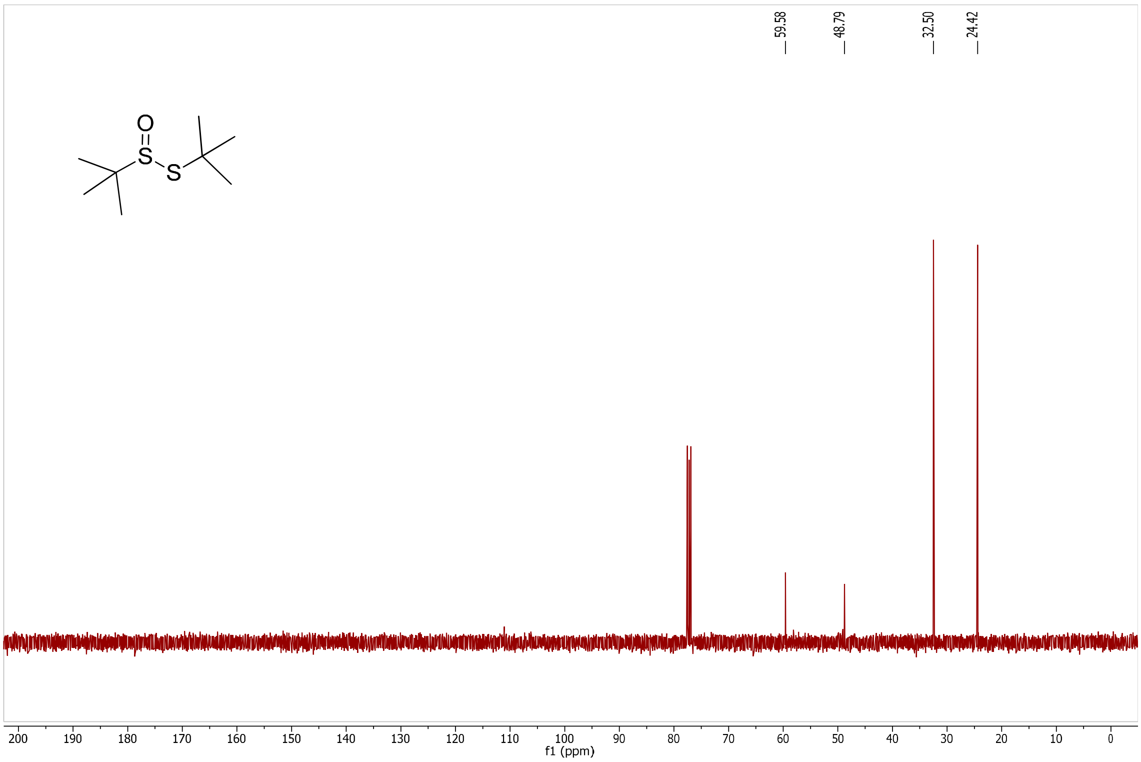

Figure S11. ^{13}C-NMR spectrum of thiolsulfinate **2f** ($CDCl_3$).

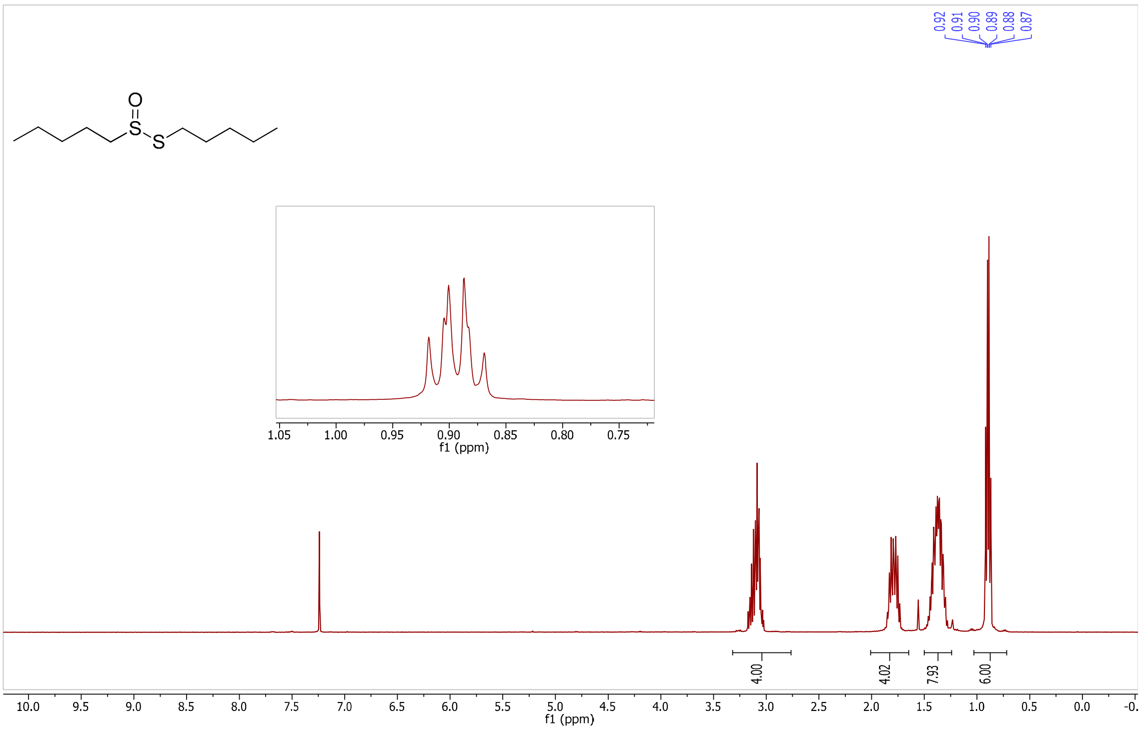

Figure S12. ^{1}H-NMR spectrum of thiolsulfinate **2g** ($CDCl_3$).

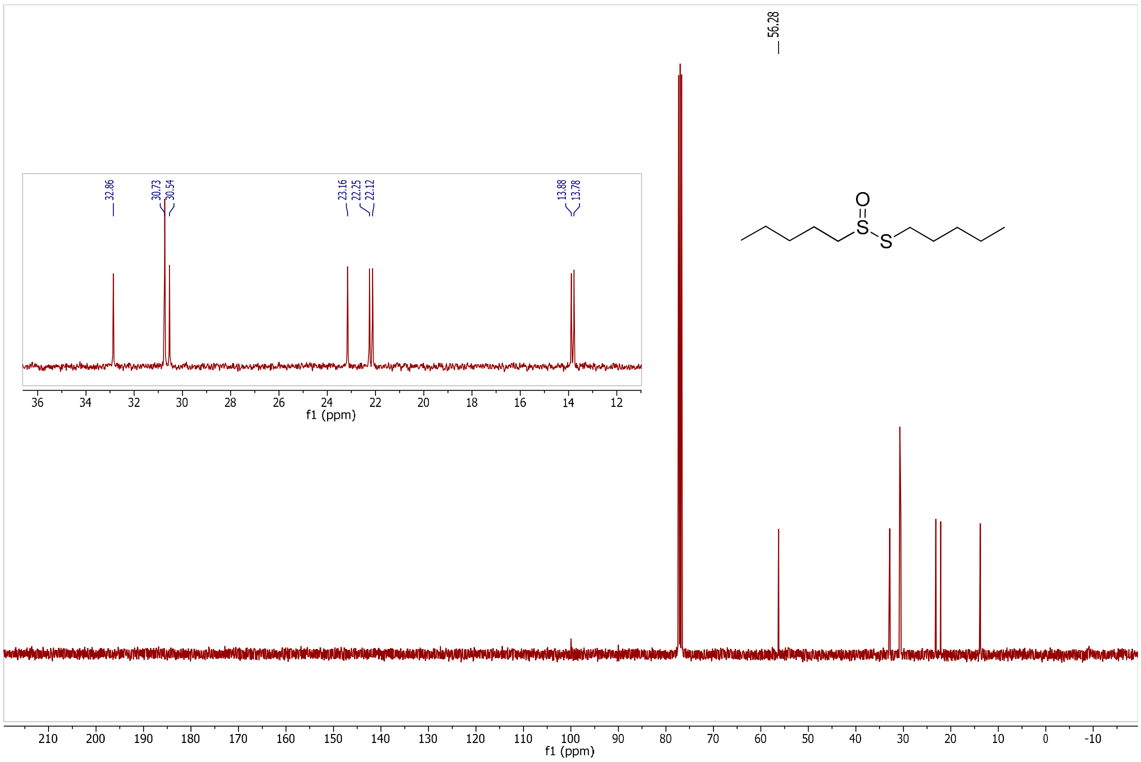

Figure S13. ^{13}C-NMR spectrum of thiolsulfinate **2g** ($CDCl_3$).

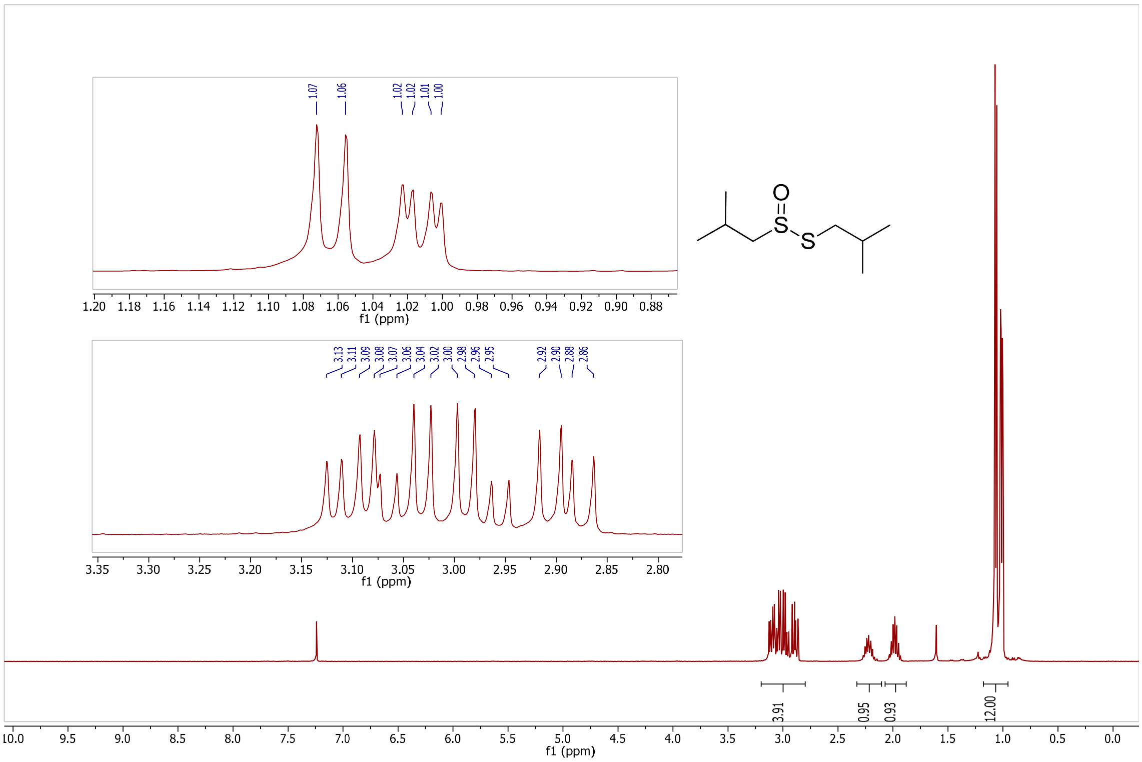

Figure S14. ^{1}H-NMR spectrum of thiolsulfinate **2h** ($CDCl_3$).

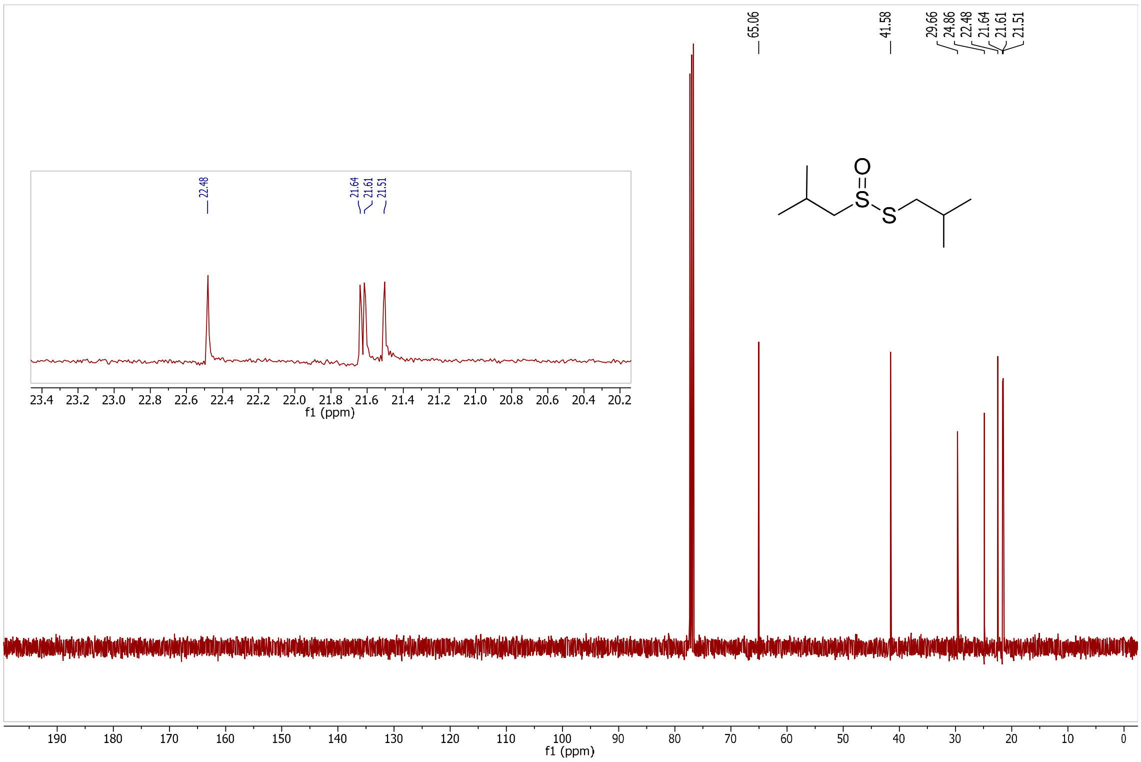

Figure S15. ^{13}C-NMR spectrum of thiolsulfinate **2h** ($CDCl_3$).

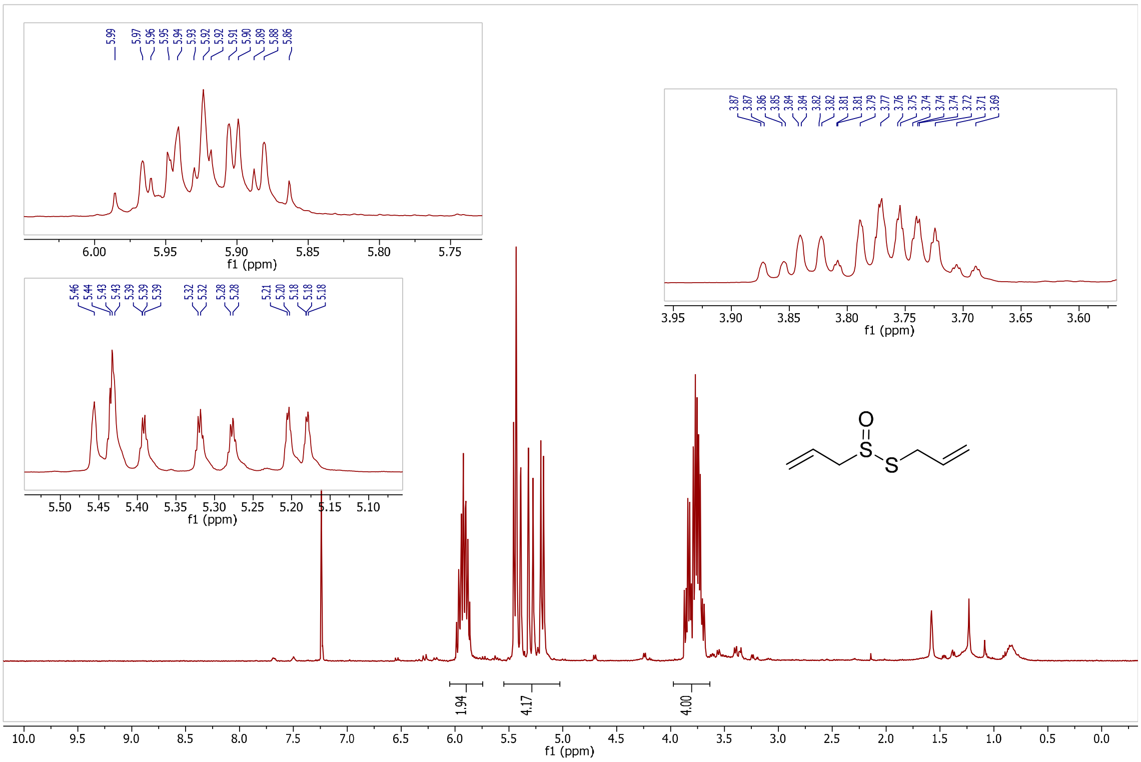

Figure S16. ^{1}H-NMR spectrum of thiolsulfinate **2i** ($CDCl_3$).

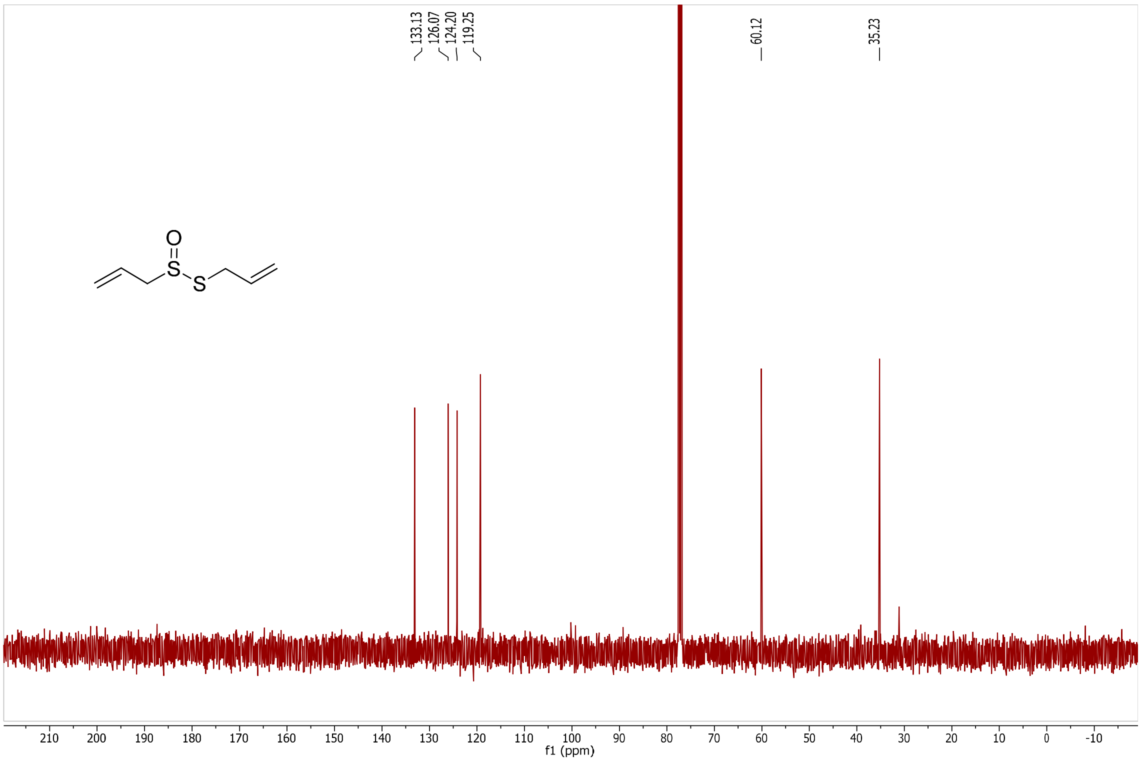

Figure S17. ^{13}C-NMR spectrum of thiolsulfinate **2i** ($CDCl_3$).

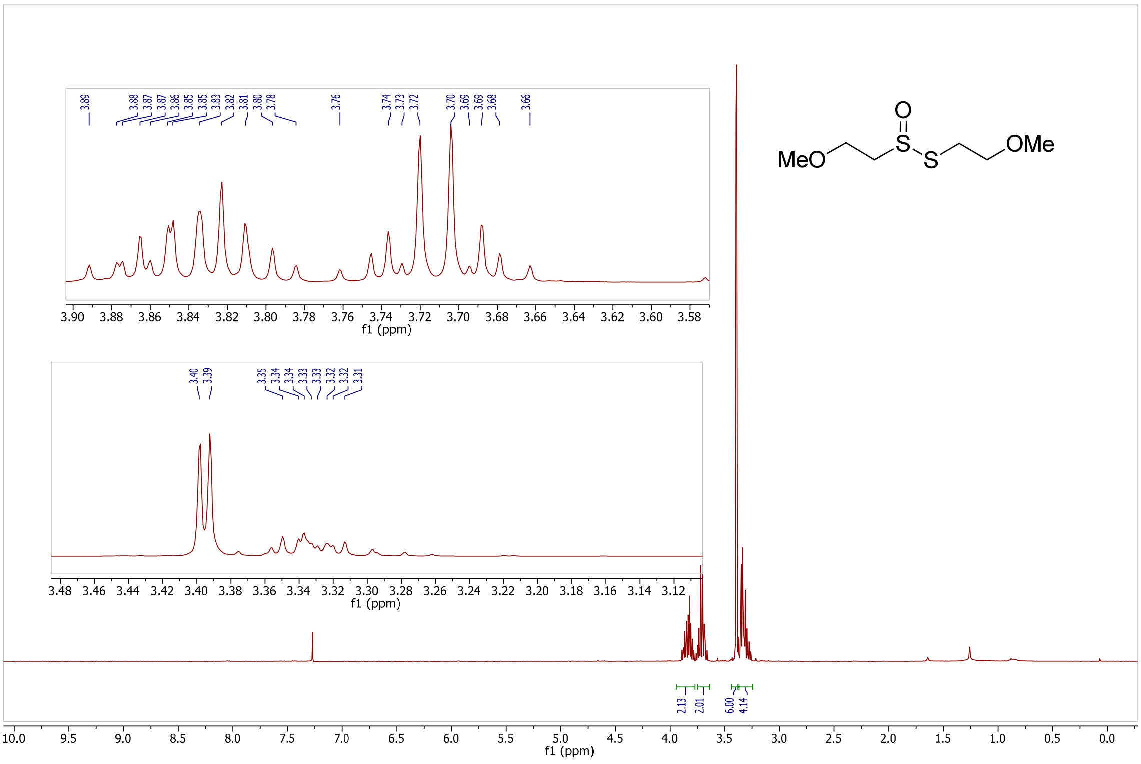

Figure S18. ^{1}H-NMR spectrum of thiolsulfinate **2j** ($CDCl_3$).

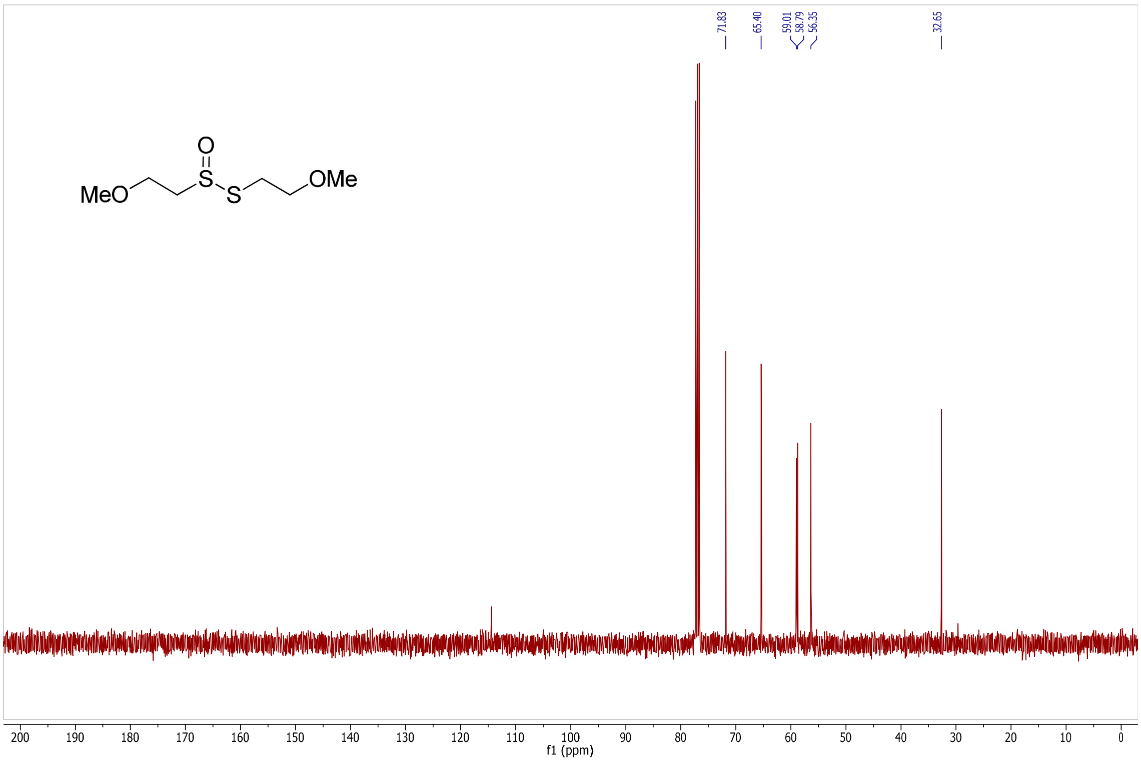

Figure S19. ^{13}C-NMR spectrum of thiolsulfinate **2j** ($CDCl_3$).

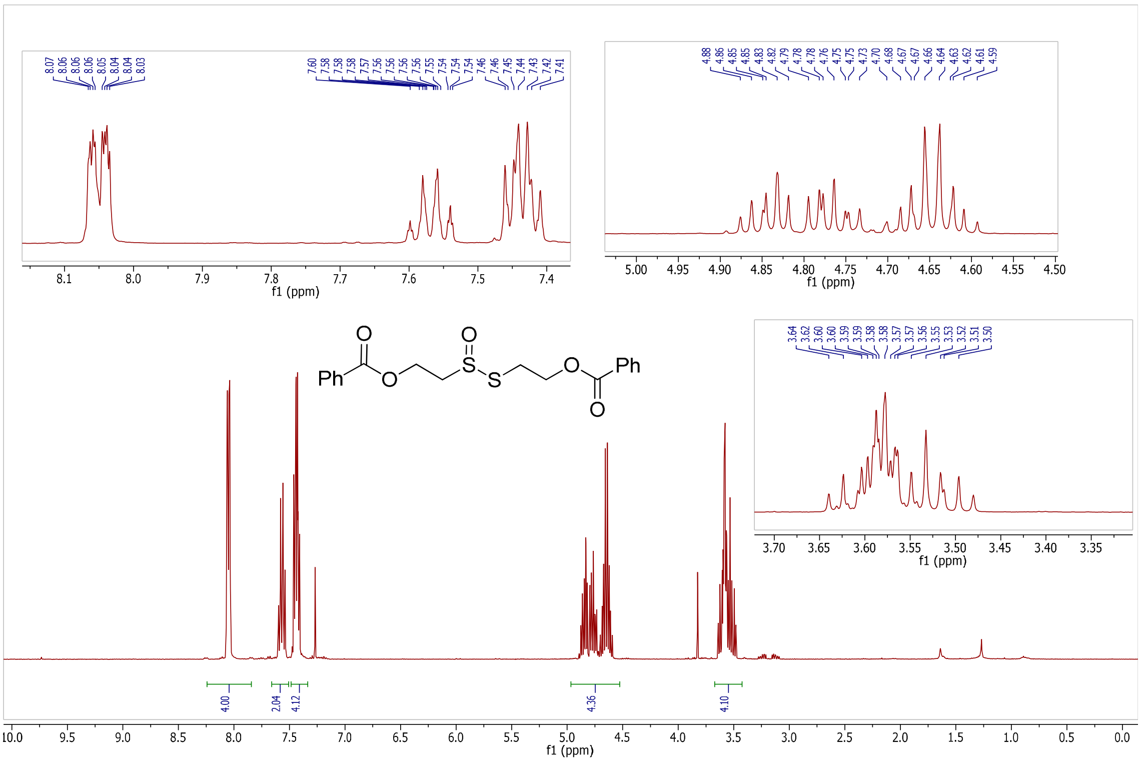

Figure S20. ^{1}H-NMR spectrum of thiolsulfinate **2k** ($CDCl_3$).

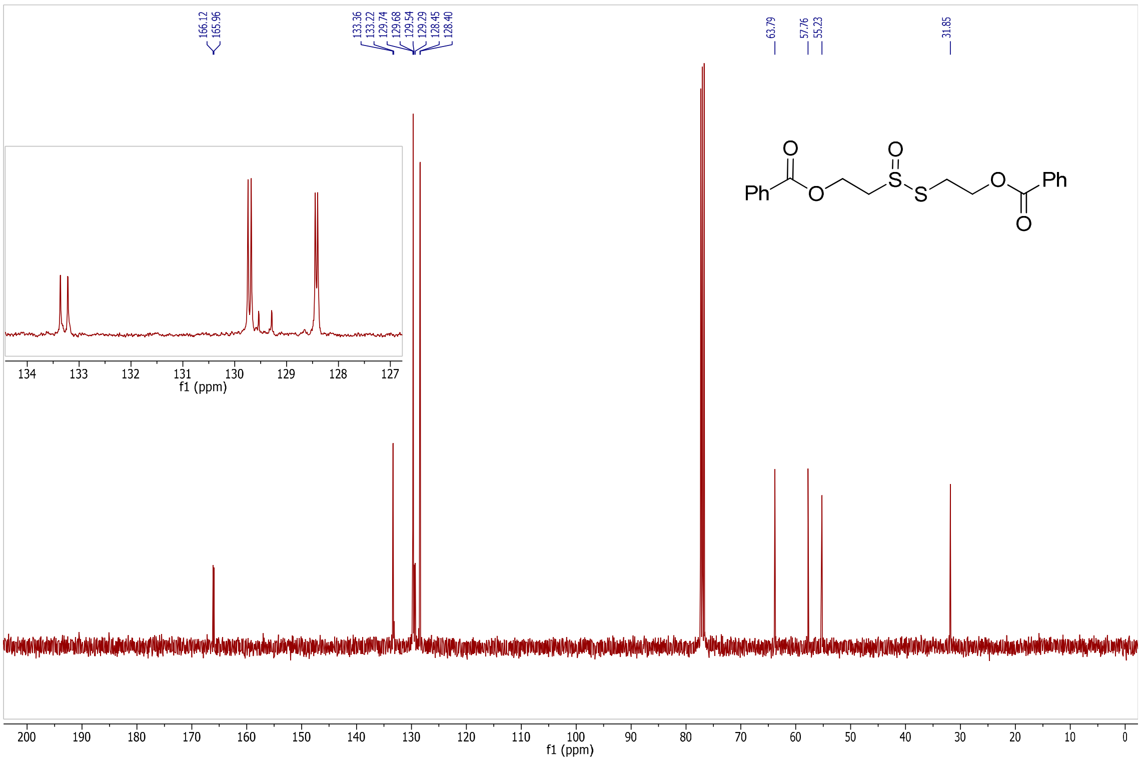

Figure S21. ^{13}C-NMR spectrum of thiolsulfinate **2k** ($CDCl_3$).

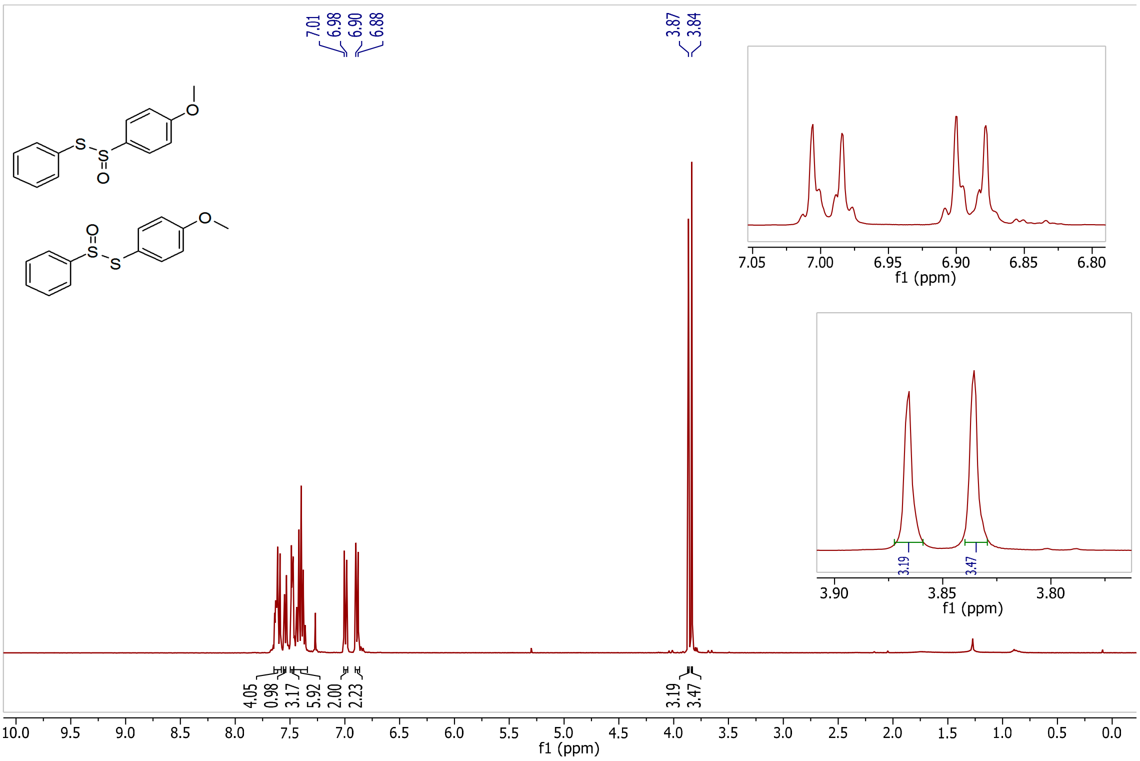

Figure S22. ^{1}H-NMR spectrum of thiolsulfinates **4a** and **5a** ($CDCl_3$).

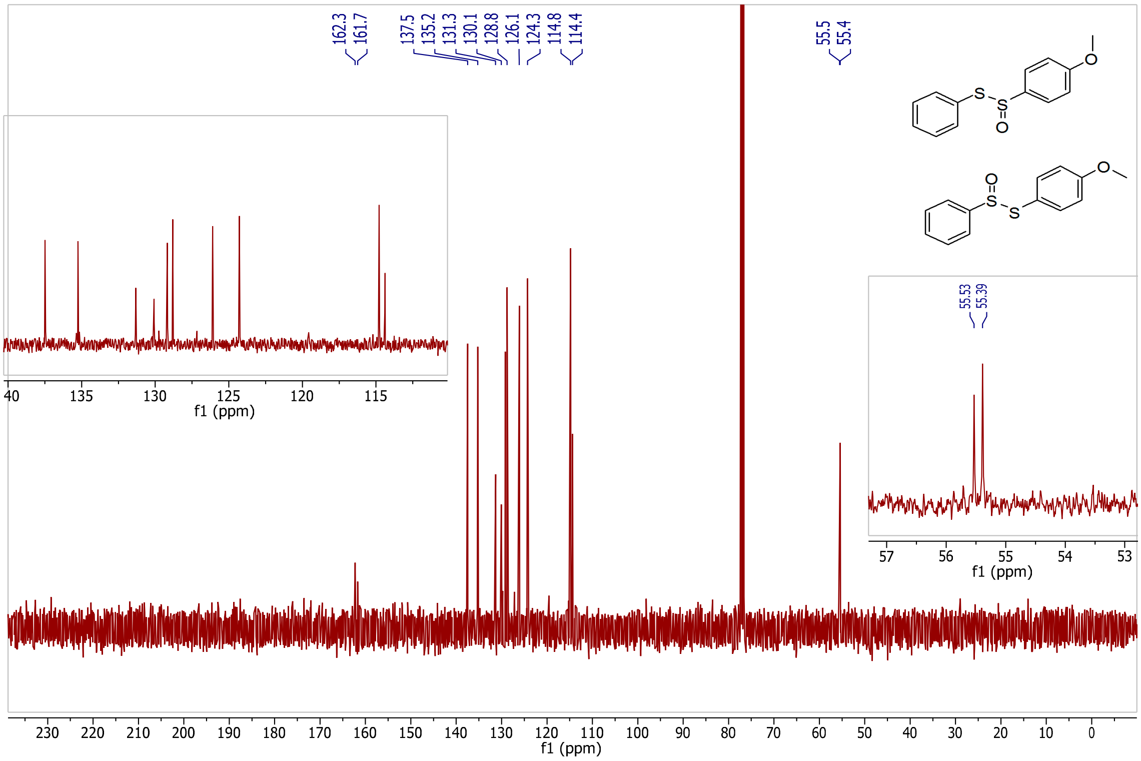

Figure S23. ^{13}C-NMR spectrum of thiolsulfinates **4a** and **5a** ($CDCl_3$).

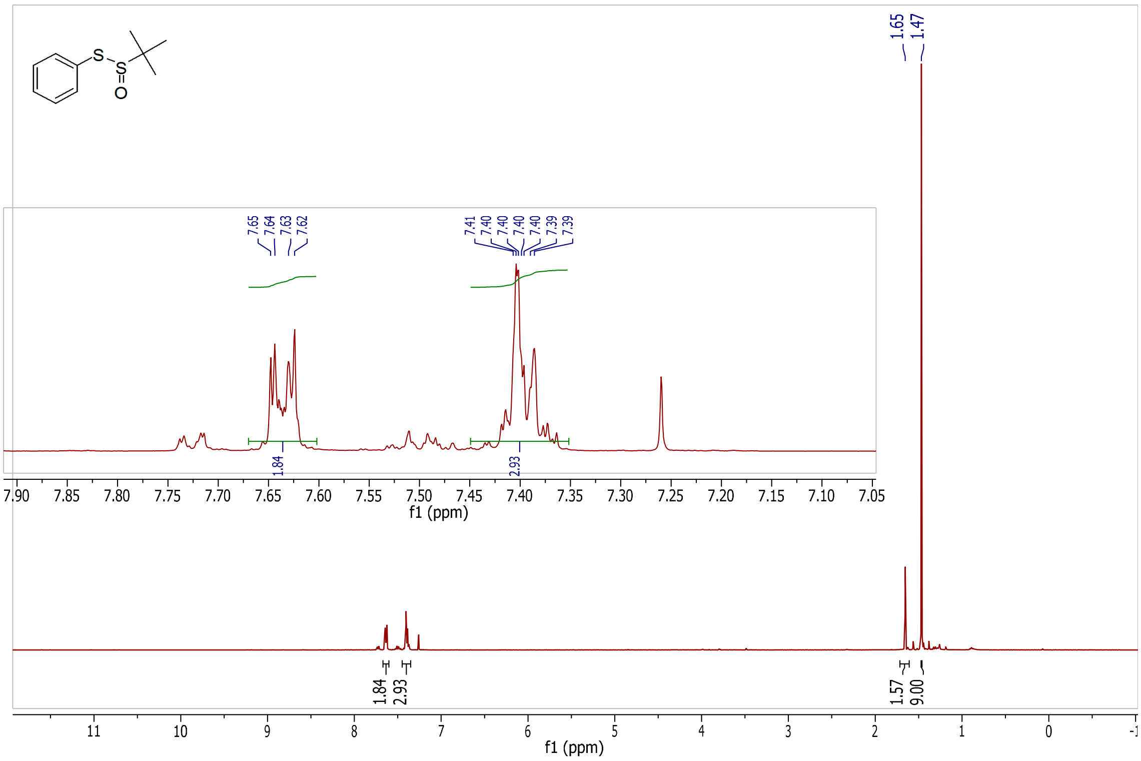

Figure S24. ^{1}H-NMR spectrum of thiolsulfinates **4b** and **5b** ($CDCl_3$).

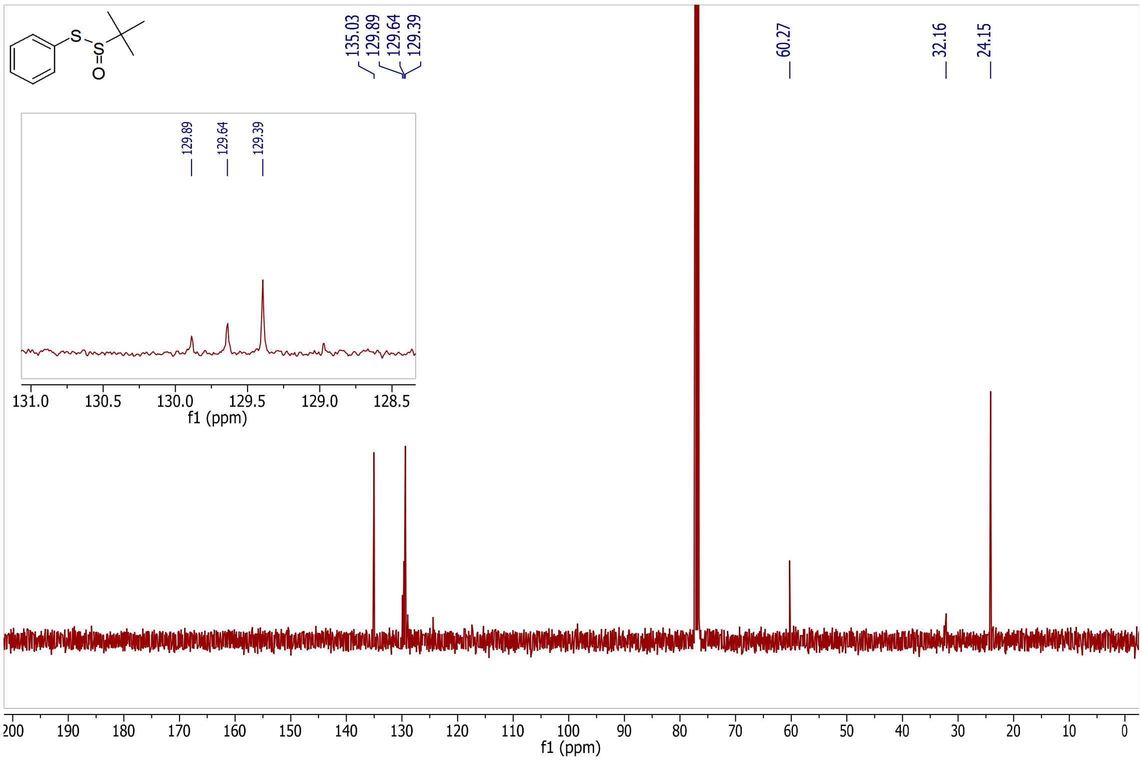

Figure S25. ^{13}C-NMR spectrum of thiolsulfinates **4b** and **5b** ($CDCl_3$).

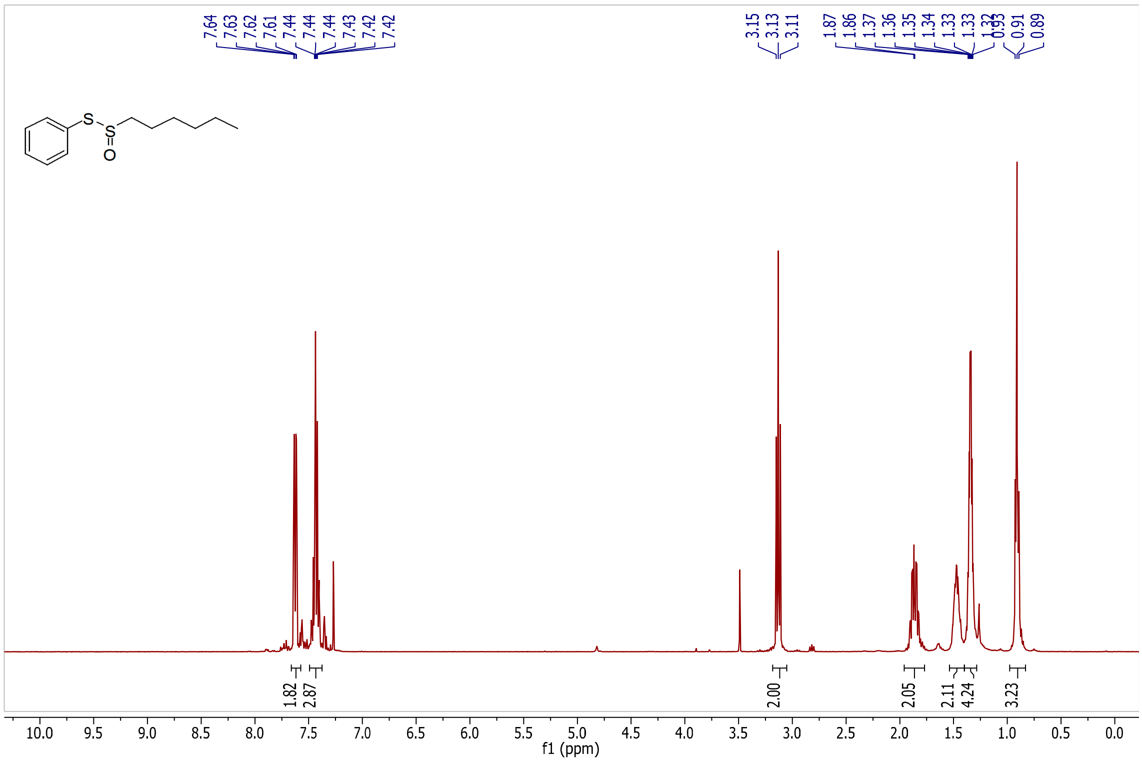

Figure S26. ^{1}H-NMR spectrum of thiolsulfinate **4c** ($CDCl_3$).

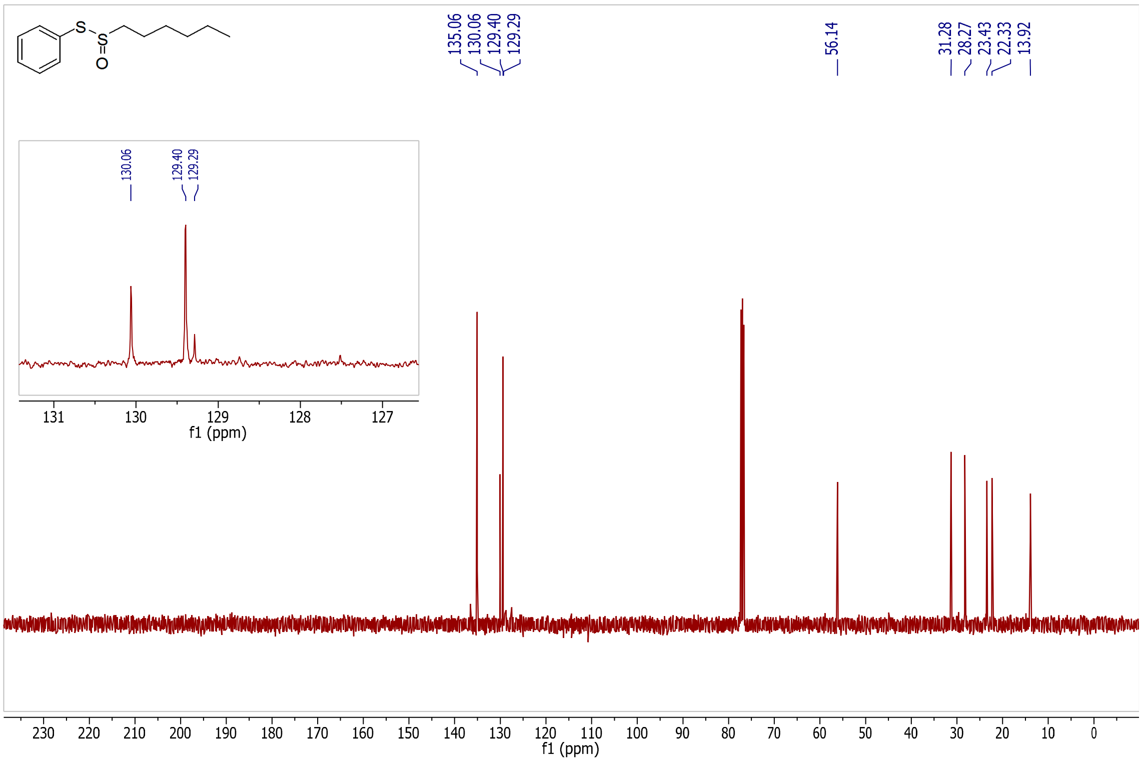

Figure S27. ^{13}C-NMR spectrum of thiolsulfinate **4c** ($CDCl_3$).

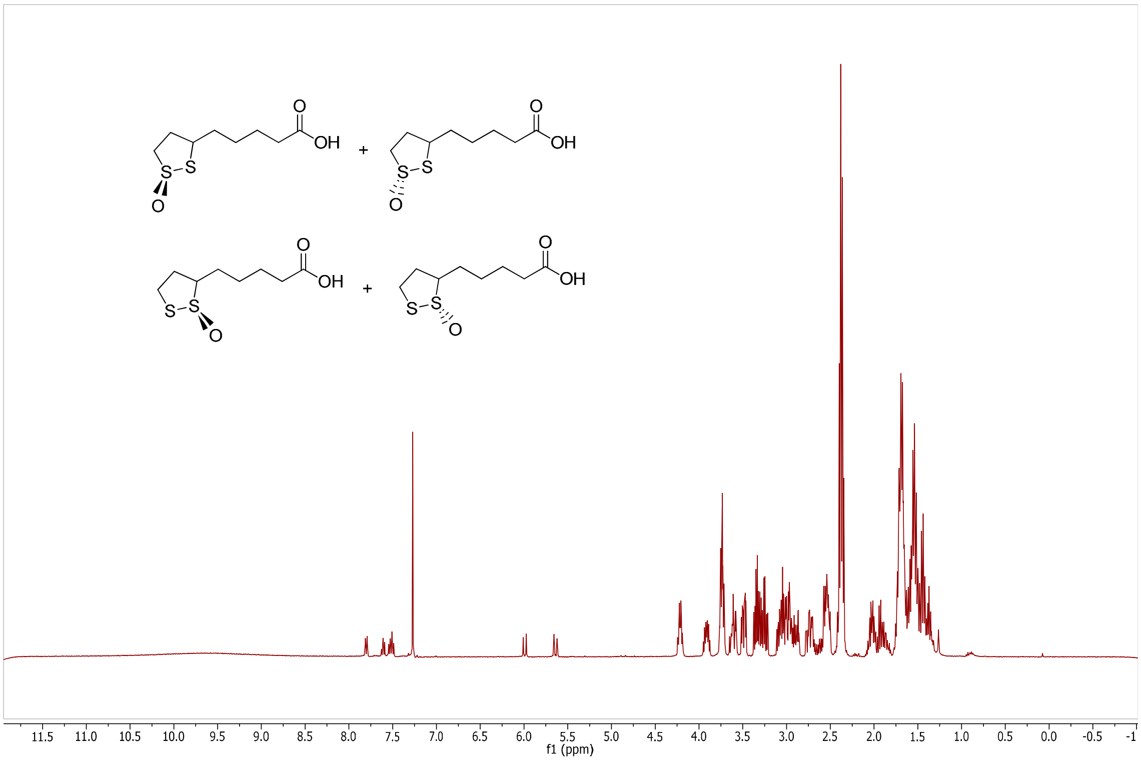

Figure S28. ^{1}H-NMR full spectrum of thiolsulfinates **8–11** from (±)-lipoic acid ($CDCl_3$).

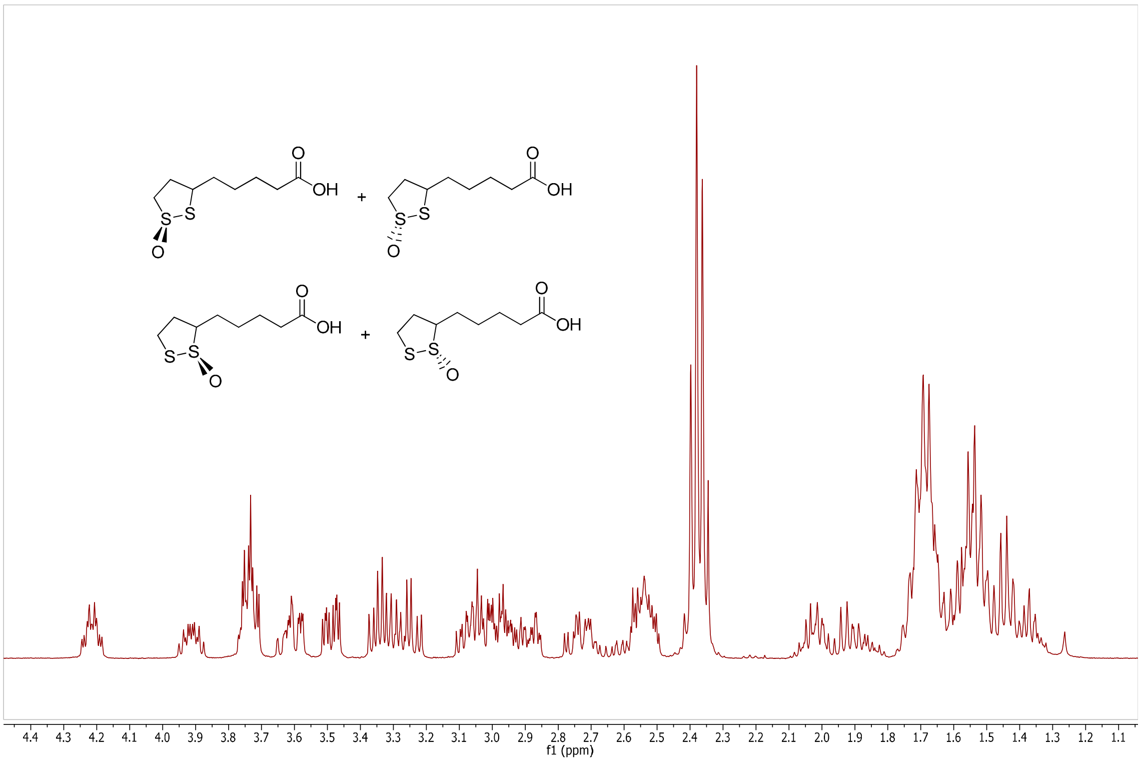

Figure S29. ^{1}H-NMR expanded spectrum of thiolsulfinates **8–11** from (±)-lipoic acid ($CDCl_3$).

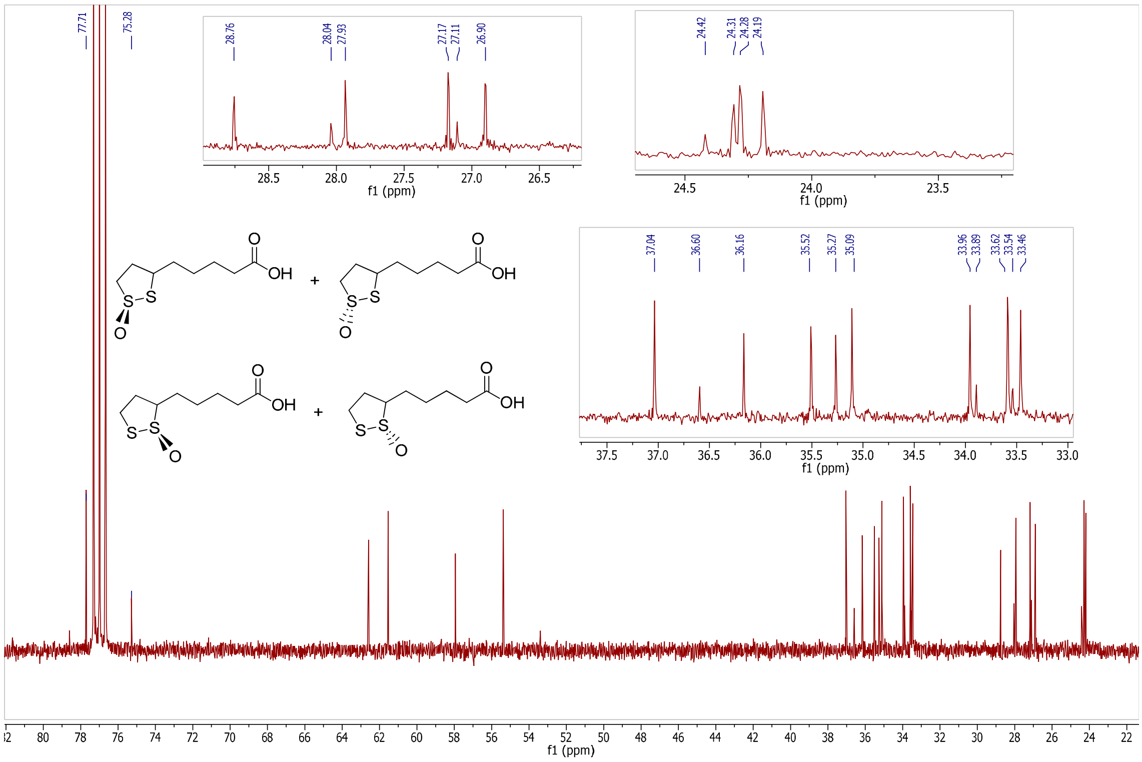

Figure S30. ^{13}C-NMR expanded spectrum of thiolsulfinates **8–11** from (±)-lipoic acid ($CDCl_3$).

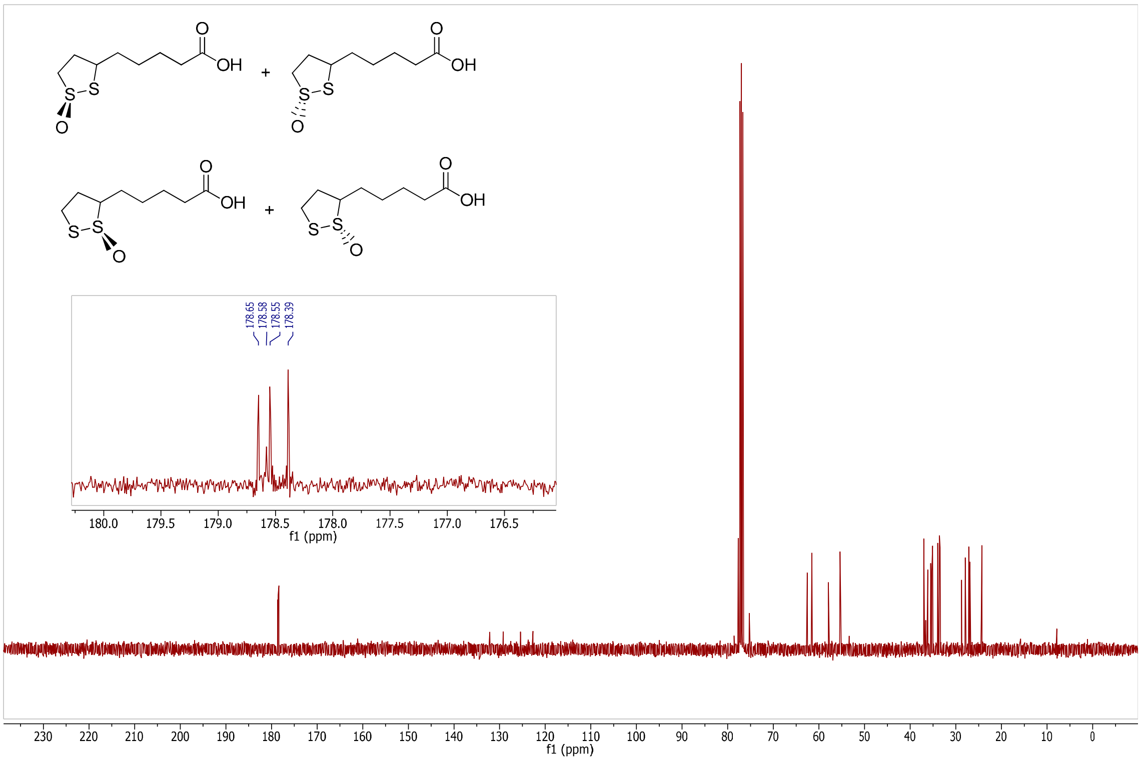

Figure S31. ^{13}C-NMR full spectrum of thiolsulfinates **8–11** from (±)-lipoic acid ($CDCl_3$).

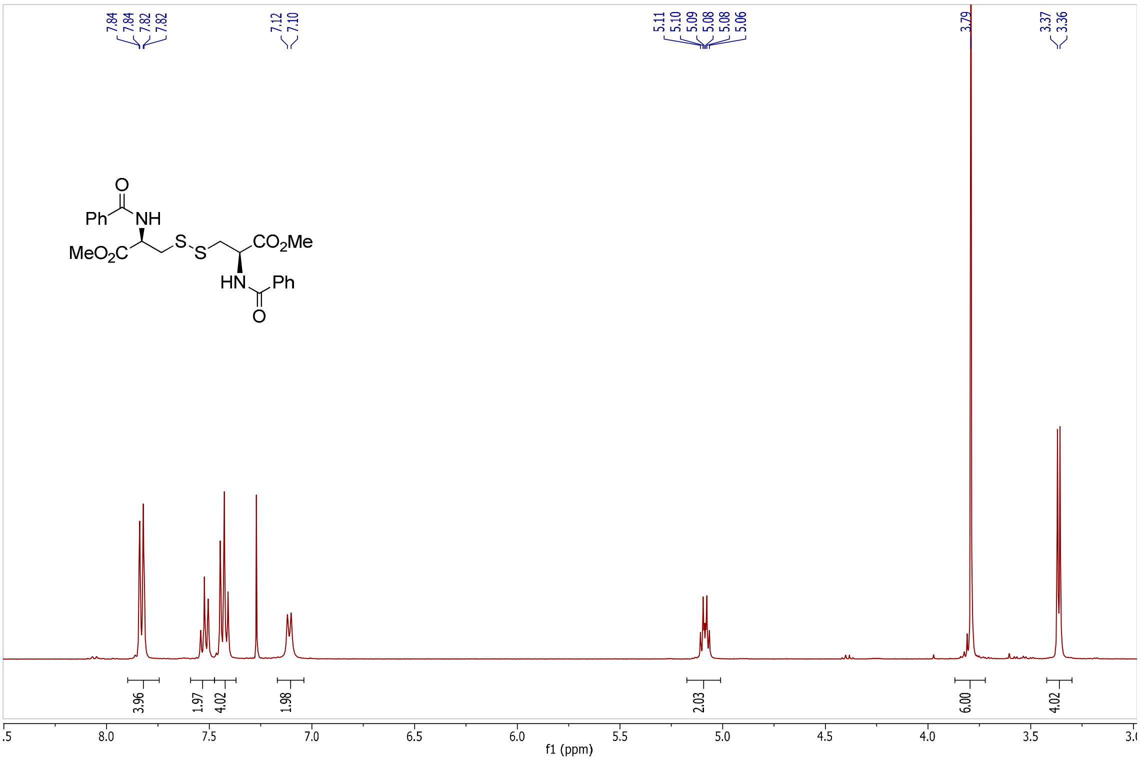

Figure S32. ^{1}H-NMR spectrum of cystine derivative **12** ($CDCl_3$).

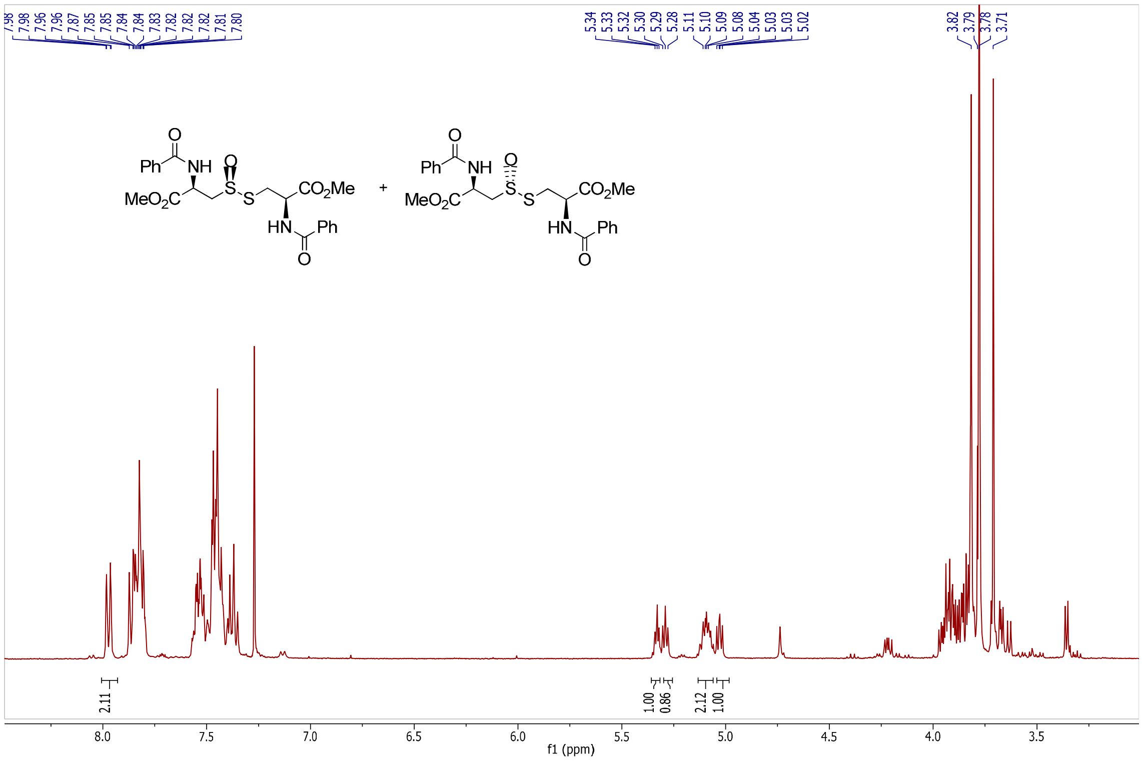

Figure S33. ^{1}H-NMR spectrum of oxidation products of cystine derivative **12** ($CDCl_3$).
